# Long-term Outcome of Adult Patients With Membranous Nephropathy Treated With Rituximab

**DOI:** 10.1016/j.ekir.2025.05.013

**Published:** 2025-05-21

**Authors:** Maria J. Vargas-Brochero, Elisabeth Lafaut, Yeshwanter Radhakrishnan, Ilario Russo, Sanjeev Sethi, Ladan Zand, Daniela Valencia, Miriam Machado, Maria Jose Soler, Anila Cara, Gian Marco Berti, Daniel C. Cattran, Fernando C. Fervenza

**Affiliations:** 1 Division of Nephrology and Hypertension, Department of Medicine, Mayo Clinic College of Medicine and Science, Rochester, Minnesota, USA; 2 Department of Internal Medicine, Ghent University, Ghent, Belgium; 3 Department of Precision and Regenerative Medicine and Ionian Area, University of Bari Aldo Moro, Bari, Italy; 4 Department of Laboratory Medicine and Pathology, Mayo Clinic, Rochester, Minnesota, USA; 5 Department of Nephrology, Centro de Referencia en Enfermedad Glomerular Compleja del Sistema Nacional de Salud, Vall d’Hebron University Hospital, Vall d’Hebron Institute of Research, Barcelona, Spain; 6 Division of Nephrology, University of Toronto, Ontario, Canada

**Keywords:** long-term outcome, membranous nephropathy, remission, rituximab

## Abstract

**Introduction:**

Rituximab (RTX) therapy has become the standard of care for treatment of membranous nephropathy (MN). However, data on hard outcomes such as end-stage kidney disease (ESKD) and loss of estimated glomerular filtration rate (eGFR), are lacking.

**Methods:**

This was a retrospective study on all patients with MN treated with RTX between January 2000 and December 2022. The primary outcomes were ESKD and eGFR loss > 50%. Clinical outcomes were complete remission (CR), partial remission (PR) (reduction in baseline proteinuria ≥ 50% and proteinuria ≤ 3.5 g/24 h), and immunological remission (IR) (serum antiphospholipase A receptor antibody [PLA2R-Ab] depletion).

**Results:**

A total of 159 patients were included (75.5% male, 87.4% White, median age: 58 years); 52.8% had previous immunosuppression (IS). Baseline serum creatinine was 1.50 (1.1–1.9) mg/dl, eGFR was 54.6 (37.4–72.5) ml/min per 1.73 m^2^, proteinuria was 9.2 (6.7–11.9) g/24 h, and serum albumin was 2.7 (2.2–3.2) g/dl; Of the patients, 108 (75.5%) had PLA2R-Ab–associated MN (PLA2R-MN); and 140 of 159 (88.1%) attained CR or PR. Median (interquartile range [IQR]) time to CR and PR were 22.6 (15.5–37.4) and 6.8 (3.6–12.1) months, respectively. Failure to respond to RTX was observed in 11.9% of patients. Previous IS and interstitial fibrosis/tubular atrophy (IFTA) ≥ 25% were independent factors associated with failure to respond to RTX. Patients treated only with RTX with a median follow-up of 62.6 months; 7 of 159 (4.4%) developed ESKD with an estimated renal survival of 97% (95% confidence interval [CI]: 94%–100%) and 95.4% (95% CI: 91.2%–99%) at 5 and 10 years, respectively.

**Conclusion:**

RTX treatment is associated with excellent long-term renal survival that compares favorably with historical survival rates using the cyclic corticosteroids/cyclophosphamide regimen.

## Graphical abstract

**Figure:**
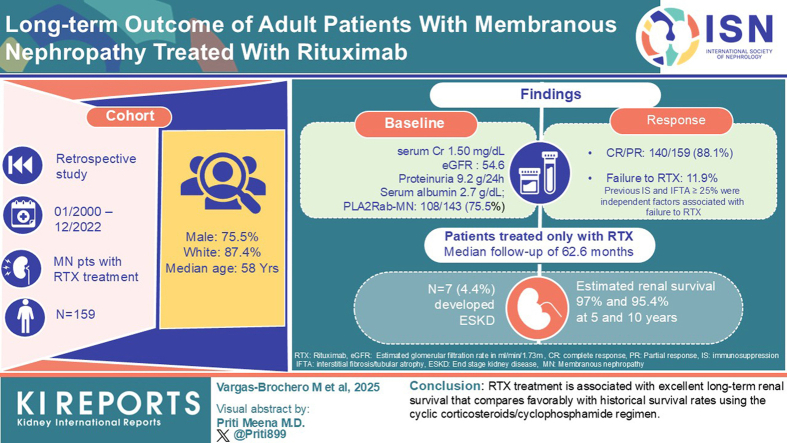

MN is a pattern of glomerular injury caused by autoantibodies against specific glomerular antigens and is currently classified according to the target antigen involved.^1^ The M-type PLA2R is the target antigen in up to 70% of MN cases.^2^ The insight that MN is a renal-limited autoimmune disease resulting in circulating autoantibodies against target antigens^3^ has given impetus to anti–B-cell–directed therapies to eliminate autoantibody production.^4^ Therefore, anti-CD20 treatment, most commonly with RTX, has become the current standard of care in MN.^5^, ^6^, ^7^

Recent randomized trials such as the Evaluate RTX Treatment for Idiopathic MN trial,^8^ MN Trial of RTX,^6^ RTX or Cyclophosphamide in the Treatment of MN^9^ have shown that RTX compares favorably with cyclosporine and cyclical cyclophosphamide/glucocorticoids with respect to proteinuria reduction and immunological remission (IR).^10^ However, follow-up duration in these trials is 17, 24, and 24 months, respectively, with no long-term data available.^6^^,^^8^^,^^9^ Hard clinical end points, such as the development of ESKD, were examined in only 1 prospective study of 100 patients with a median follow-up of 29 months that had 4% incidence of ESKD.^11^ Furthermore, previous studies using cyclic corticosteroids/cyclophosphamide regimens have not reported on eGFR status over time. Considering that kidney survival with preservation of eGFR is the primary goal of treatment,^12^ the aim of this study was to evaluate the long-term outcomes in terms of ESKD progression and eGFR preservation in patients with MN treated with RTX.

## Methods

### Patient Cohort

All consecutive adult patients with biopsy-proven MN in the native kidney seen at the Mayo Clinic from January 1, 2000 to December 31, 2022 and treated with RTX were included in the study. RTX treatment was at the discretion of the treating physician. Patients with previous IS regimens were included if they had > 6 months without cyclophosphamide, and > 1 month without calcineurin inhibitors, and had stable or increasing proteinuria. Patients with proteinuria < 3.5 g/24 h, secondary MN, superimposed primary glomerulopathies, RTX used as a sparing calcineurin sparing agent, or ESKD at the time of RTX initiation were excluded.

### Data Collection

The study was approved by the Mayo Clinic Institutional Review Board and conducted in accordance with the Declaration of Helsinki, Mayo Clinic institutional policies, and regulations for the protection of human subjects.

Data were retrospectively abstracted from electronic medical records. Patient demographic and clinical characteristics were collected at baseline. Baseline was defined as the time of the first RTX treatment. Laboratory values, including proteinuria and eGFR, were obtained at baseline and follow-up yearly. Anti- PLA2R-Ab titers were recorded when available. Missing data were addressed by reporting the available data for each variable.

### Definitions and Outcomes

eGFR was estimated using the Chronic Kidney Disease-Epidemiology Collaboration creatinine equation (2021). CR was defined as proteinuria ≤ 0.3 g/24 h and serum albumin ≥ 3.5 g/dl. PR was defined as a reduction in baseline proteinuria ≥ 50% and proteinuria ≤ 3.5 g/24 h but > 0.3 g/24 h.^12^ Reduction of proteinuria (RP) was defined as a reduction in baseline proteinuria ≥ 50%, but not CR or PR.^11^^,^^13^ Failure to respond to RTX was defined as patients who did not achieve any response (CR or PR) during follow-up or needed to change therapy to another IS. ESKD was defined as the first occurrence of patient-initiated dialysis or kidney transplant. Worsening of kidney function was defined as >50% reduction in baseline eGFR. Relapse was defined as developing proteinuria > 3.5 g/24 h following CR or PR. Anti-PLA2R-Ab were evaluated using a standardized commercial enzyme-linked immunosorbent assay (Euroimmun, Lubeck, Germany)^14^ and indirect immunofluorescence testing. PLA2R-MN was defined as having positive PLA2R-Ab, defined as PLA2R-Ab > 14 RU/ml and positive indirect immunofluorescence testing, at any time during the follow-up. IR was defined as serum PLA2R-Ab < 2 RU/ml or PLA2R-Ab levels < 14 RU/ml with negative indirect immunofluorescence testing.^5^^,^^15^

### RTX Treatment Regimen

The therapeutic regimen used for RTX was RTX 1 g (day 1/day 14), 375 mg/m^2^ every 4 weeks, or a single 1 g dose. All patients were premedicated with oral acetaminophen (1000 mg), diphenhydramine hydrochloride (50 mg), and methylprednisolone (100 mg, i.v.) before the first RTX infusion. The use of prophylaxis for *Pneumocystis jirovecii* was also evaluated.

### Statistical Methods

Continuous variables were summarized using mean (SD) for normally distributed data and median (IQR) for nonnormally distributed data; for categorical variables, data were summarized using *n* (%). Pearson’s chi-square test was used to compare categorical variables between groups; for comparison of continuous variables between groups, *t* test, Mann–Whitney U test, and Kruskal–Wallis test were used depending on the number of compared groups and the distribution of variables. Changes in laboratory values over time were analyzed using the paired *t* test and the signed-rank test, depending on the distribution.

Time-to-event analysis using the Kaplan-Meier method was used to assess the outcomes of ESKD, worsening kidney function, and failure to respond to RTX. Renal survival (i.e., ESKD) and failure to respond to RTX were evaluated in the entire cohort with patients being censored at the last follow-up. To evaluate renal outcomes in patients treated exclusively with RTX after enrollment, renal survival (i.e., event ESKD), and a composite outcome of ESKD and/or worsening kidney function, as well as failure to respond to RTX and worsening kidney function, were evaluated with patients censored at the time of the last follow-up or changed treatment regimen, whichever occurred first. In addition, the evaluation of renal survival in patients who responded to RTX is reported. Comparisons across groups were evaluated using the log-rank test. Cox models were fitted to compute hazard ratios (HRs) and 95% CIs for predictive factors for failure to respond to RTX, ESKD, and worsening kidney function. Sensitivity analysis was conducted using baseline characteristics and outcomes (clinical response and renal survival) between patients with nephrotic syndrome versus those with normal serum albumin.

Secondary outcomes included CR, PR, and RP annual percentages. The time to the first clinical response (CR, PR, or RP), IR, and relapse was described in months. Subgroup analyses of patients with focal segmental glomerulosclerosis (FSGS) lesions in the kidney biopsy were performed to account for potential confounding variables. Baseline anti-PLA2R titers were analyzed using the receiver operating characteristic curve to find the optimal cutoff value for stratifying patients.

The safety outcomes were infections, thromboembolic events, and death, which were summarized using *n* (%). The occurrence of cancer was described in terms of incidence rate. We calculated the annual incidence rate as a measure of the number of new cases (“incidence”) per unit of time (“rate”), in this case 1000 person-years. We included all types of cancers diagnosed after a biopsy confirmation. The observation period was between RTX initiation and the date of cancer diagnosis, death, ESKD (transplant and dialysis), or last follow-up, whichever came first.

*P*-values < 0.05 were considered statistically significant. All statistical analyses were performed using BlueSky Statistics software v. 10.3 (BlueSky Statistics LLC, Chicago, IL).

## Results

### Baseline Clinical Characteristics

A total of 249 patients were screened, of which 159 met the inclusion criteria (Supplementary Figure S1). Details of the baseline clinical characteristics are presented in Table 1. Median age (IQR) at the time of RTX initiation was 58 (47–65) years; 120 patients (75.5%) were male, and 139 patients (87.4%) were White, with the second most common race being Hispanic with 11 patients (6.9%).

**Table 1:** Baseline patient characteristics

Baseline patient characteristic	Total *N* = 159
Demographics	
Age (yrs)	58 (47–65)
Male sex	120 (75.5%)
White race	139 (87.4%)
Body mass index (kg/m^2^)	29.3 (26.5–33.1)
Clinical characteristics	
Hypertension	92 (57.9%)
Diabetes mellitus	18 (11.3%)
History of autoimmune disease	5 (3.1%)
Previous IS	84 (52.8%)
Calcineurin inhibitor alone	37 (23.2%)
Cyclic CYC/steroids	16 (10.1%)
Cyclosporine+ CYC/steroids	10 (6.4%)
Cyclic CYC/steroids and tacrolimus	2 (1.3%)
Steroids	8 (5.0%)
MMF	7 (4.4%)
MMF and steroids	1 (0.6%)
ACTH	1 (0.6%)
ACTH and cyclosporine	1 (0.6%)
Tacrolimus and MMF	1 (0.6%)
Relapse after previous IS	39 (24.5%)
No response to previous IS	45 (28.3%)
Systolic blood pressure (mm Hg)	126 (116–136)
Diastolic blood pressure (mm Hg)	74 (69–82)
Serum creatinine (mg/dl)	1.50 (1.1–1.9)
eGFR (ml/min per 1.73 m^2^)	54.6 (37.4–72.5)
Proteinuria (g/24 h)	9.2 (6.7–11.9)
Serum albumin (g/dl)	2.7 (2.2–3.2)
Nephrotic syndrome	136 (85.5%)
Hematuria	77 (48.4%)
Creatinine clearance 24 h ml/min/BSA	60 (49,85)^a^
Edema	145 (91.2%)
Hemoglobin (g/dl)	12.7 (1.8)^e^
Cholesterol (mg/dl)	246 (203–332)^b^
LDL cholesterol (mg/dl)	149 (108–213)^c^
Triglycerides (mg/dl)	196 (136–267)^b^
PLA2R associated MN	*N* = 143; 108 (75.5%)
Anti PLA2R antibody titers available at the time of RTX initiation (RU/ml),] in PLA2R associated MN	84 (34–180.5)^d^
Biopsy characteristics	
Interstitial fibrosis / tubular atrophy ≥ 25%	22 (14.2%)^e^
Interstitial fibrosis / tubular atrophy	5 (4,15)^e^
Percentage of glomerulosclerosis %	4.3 (0–9.1)^e^
FSGS lesion	36 (23.2%)^e^
Diffuse foot process effacement	124 (94.7%)^f^
Diabetic nephropathy	3 (1.9%)

ACTH, adrenocorticotropic hormone; BSA, body surface area; CYC, cyclophosphamide; eGFR, estimated glomerular filtration rate; FSGS, focal segmental glomerulosclerosis; IQR, interquartile range; IS, immunosuppression; MMF, mycophenolate mofetil; MN, membranous nephropathy; PLA2R, phospholipase A2 receptor; RTX, rituximab.

Data are presented as *n* (%), mean (SD), or median [IQR].

a missing 74 values.

b missing 10 values.

c missing 20 values.

d missing 80 values.

e missing 4 values.

f missing 28 values.

A total of 84 patients (52.8%) were previously treated with previous IS, most commonly with calcineurin inhibitors in 37 patients (23.2%) and cyclic corticosteroids/cyclophosphamide regimen in 16 patients (10.1%) (Table 1). The decision to use RTX in this population was prompted by relapse in 39 patients (24.5%) and nonresponse to previous IS in 45 patients (28.3%). Among the nonresponders, 16 had been treated with calcineurin inhibitor, 5 with cyclic corticosteroids/cyclophosphamide, and 7 with both regimens. Patients were divided into 3 categories as follows: first-line RTX therapy, relapsing disease, and no response to previous IS. We observed that proteinuria levels were similar across all groups. However, the relapsing patients exhibited significantly lower eGFR (*P* = 0.003) and lower levels of anti-PLA2R-Ab (*P* = 0.003) (Supplementary Table S1).

All but 3 patients were on renin-angiotensin system inhibition at baseline. Median (IQR) systolic and diastolic blood pressure was 126 (116–136) mm Hg and 74 (69–82) mm Hg. Median [IQR] serum creatinine was 1.50 (1.1–1.9) mg/dl, and median eGFR was 54.6 (37.4–72.5) ml/min per 1.73 m^2^ with 19 patients (11.9%) having eGFR < 30 ml/min per 1.73 m^2^. Median (IQR) proteinuria was 9.2 (6.7–11.9) g/24 h, and median (IQR) serum albumin was 2.7 (2.2–3.2) g/dl, with nephrotic syndrome present in 136 patients (85.5%).

### PLA2R Status

Serum PLA2R status was available in 143 of 159 patients (89.9%) and positive in 108 of 143 patients (75.5%).

### Kidney Biopsy Characteristics

FSGS lesions were present in 36 patients (23.2%). These patients had numerically higher proteinuria at RTX initiation but similar demographic and clinical characteristics at baseline compared with those without FSGS lesions (Supplementary Table S2). A total of 22 patients (14.2%) had IFTA ≥ 25%. Diffuse foot process effacement (> 80%) was present in 124 cases (94.7%).

### Renal Outcomes: Clinical Remission and Progression to ESKD

The evolution of kidney function and proteinuria over time in patients with 5-year follow-up data available is presented in Supplementary Table S3. In this group of patients, significant declines in serum creatinine and increases in eGFR were observed over 12, 24, and 60 months compared with baseline, with the largest difference observed at 60 months. A progressive decrease in proteinuria was also observed. The improvement of eGFR was regardless of baseline eGFR.

**Figure 1:**
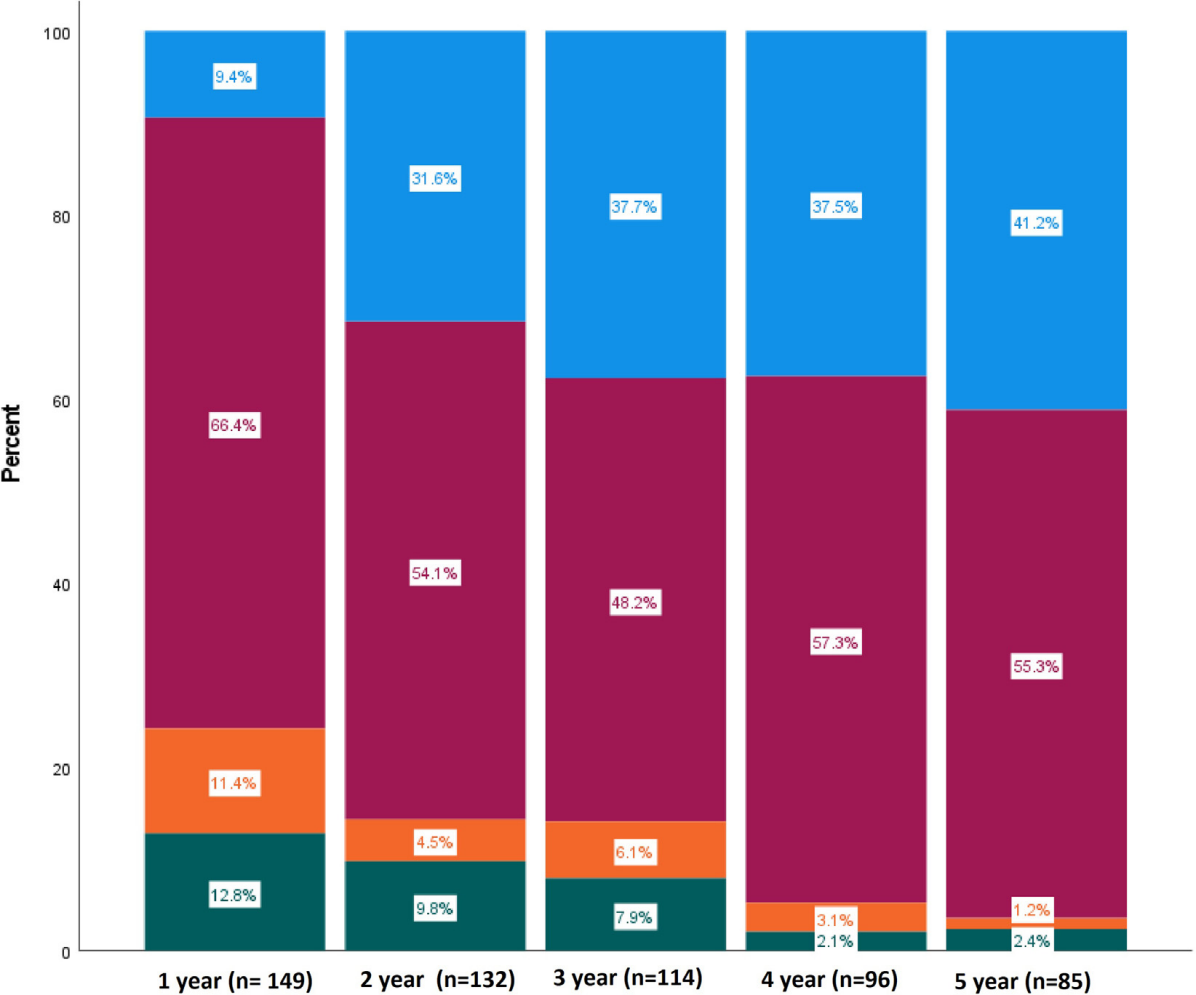
Proportion of complete remission (blue), partial remission (dark red), reduction of proteinuria ≥ 50% (light orange), and nonresponse (green) over time.

**Figure 2:**
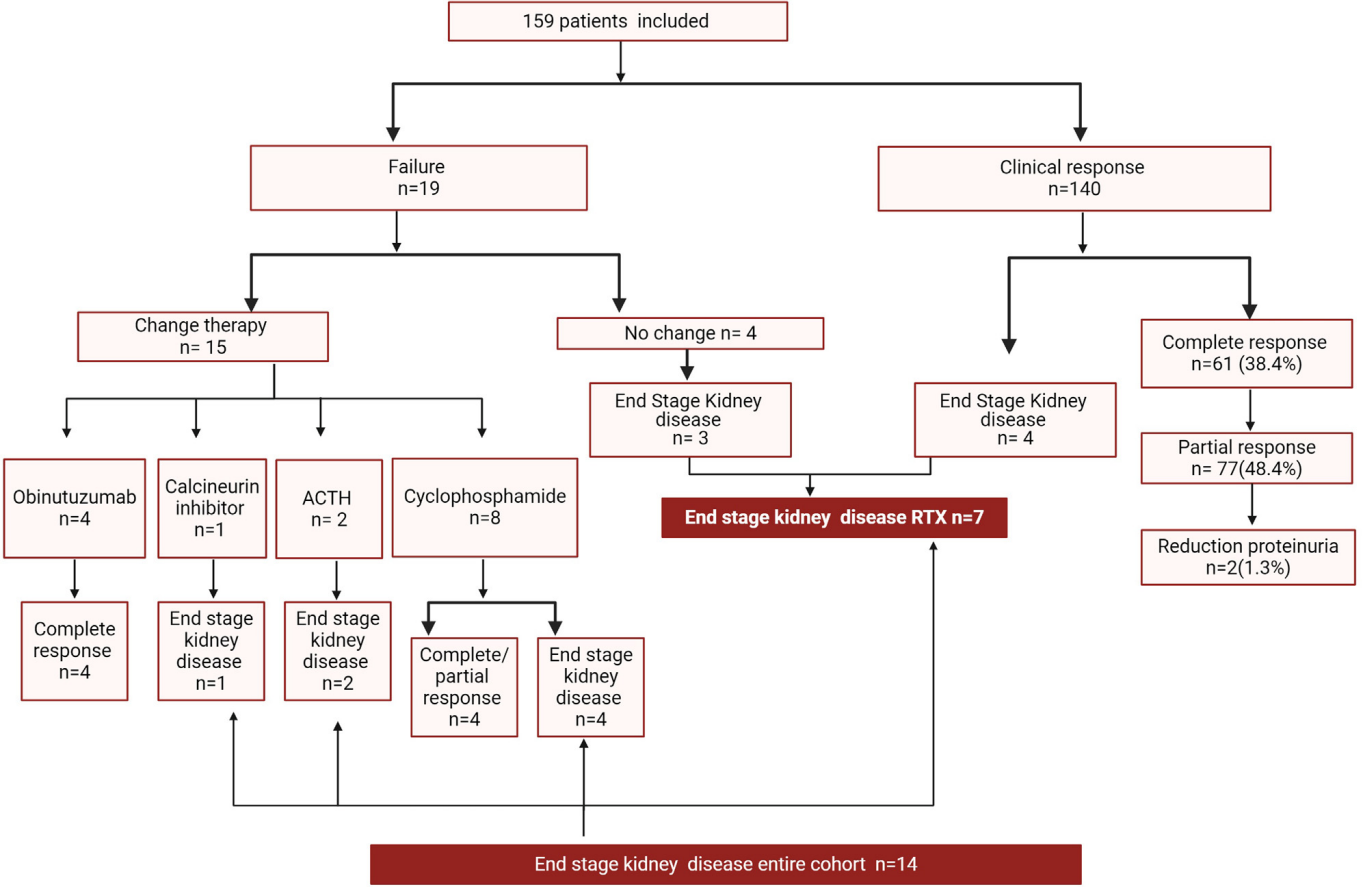
Flowchart of patients with membranous nephropathy treated with rituximab included remission, failure, and end-stage kidney disease progression with therapy changes. ACTH, adrenocorticotropic hormone; RTX, rituximab.

The proportion of patients who achieved CR increased progressively during follow-up (Figure 1). At the last follow-up, a total of 140 patients (88.1%) experienced a clinical response (CR, PR, or RP), with a median (IQR) time to response of 7.39 (3.9–12.8) months. 61 (38.4%) were in CR, 77 (48.4%) were in PR and 2 (1.2%) in RP (Figure 2).

Median time to clinical response after RTX treatment was 7.89 (95% CI: 6.8–10.6) months. The median time to first CR was 22.6 (15.5–37.4) months; the median time to first PR was 6.8 (3.6–12.1) months, and 1 patient only reached RP after 12 months. In the subset of patients with FSGS lesions, the time to PR and IR were not significantly different from those without FSGS lesions; however, the time to CR was significantly longer (Supplementary Table S2). The baseline cutoff for PLA2R-Ab titers was ≥ 116 RU/ml, which was linked to a lower chance of achieving clinical response in patients receiving RTX as first-line therapy (Supplementary Figure S2).

Among the entire cohort, 23 patients (14.5%) had normal serum albumin levels at the time of RTX initiation. Of these, 14 of 23 patients (60.8) had PLA2R-MN. Compared with the nephrotic syndrome group, these patients had a higher percentage of prior IS treatment (17 patients, 73.9%; *P* = 0.02), with 10 out of 17 (58.8%) experiencing a relapse. They also had a longer median time from diagnosis to RTX initiation (39.3 months [15.2–70.3]; *P* < 0.001), lower levels of proteinuria (6.4 [4.6–7.2] g/24 h vs. 9.9 [7.6–14.6] g/24 h; *P* < 0.001), and higher eGFR (62.4 [46.2–87.4] ml/min per 1.73 m^2^ vs. 51.8 [36.1–71.2]; *P* = 0.08). Hence, we performed a sensitivity analysis evaluating the rates of CR or PR between nephrotic patients and nonnephrotic patients, with no significant differences observed in the response rates (Supplementary Table S4). Time-to-event analysis demonstrated a significant difference in time to PR between nephrotic (8.51 months [7.0–11.73]) and nonnephrotic patients (5.49 months [3.58–9.23]) (*P* = 0.049).

Nineteen patients were categorized as therapy failure (11.9%) after a median follow-up of 61.4 [36.6–100.4] months (Supplementary Figure S3A); No significant differences in incidence of failure to respond to RTX were observed between patients with baseline eGFR < 30 ml/min per 1.73 m^2^ versus ≥ 30 ml/min per 1.73 m^2^ (log-rank *P* = 0.49), the presence versus absence of a FSGS lesion (log-rank *P* = 0.44) or baseline PLA2R-Ab levels (log-rank *P* = 0.07). There were higher failure rates in patients with IFTA ≥ 25% and previous IS (Supplementary Figure S3B and C). Of these 19 patients, 15 (78.9%) had a history of previous IS and 11 of 19 (57.8%) had PLA2R-MN with a median PLA2R-Ab of 121 (19.7–199.5) RU/ml. Four patients who failed to respond to RTX were treated with obinutuzumab, with all of them going into CR. Eight were changed to cyclophosphamide because of worsening kidney function, with 4 of them progressing to ESKD. Further details are presented in Supplementary Table S5 and Figure 2.

By the multivariate Cox regression analysis, previous IS (HR: 4.38 [95% CI: 1.44–13.35], *P* < 0.01), IFTA ≥ 25% (HR: 3.59 [95% CI: 1.34–9.55, *P* = 0.01]) and lower serum albumin (HR: 0.36 [95% CI: 0.17–0.78], *P* < 0.01) emerged as independent associated factors for failure to respond to RTX (Table 2).

**Table 2:** Univariate and multivariate Cox regression of predictive factors for failure to therapy, worsening kidney function, and composite outcome in membranous nephropathy patients treated with Rituximab

Failure to respond to RTX (*n* = 19)						
Univariate				Multivariate		
Variables	HR	95% CI	*P*-value	HR	(95% CI)	*P*-value
Male	2.81	0.65–12.18	0.16			
White	0.52	0.16–1.51	0.22			
Proteinuria (per 1 g/24 h)	1.04	0.99–1.09	0.06			
Serum albumin (per1 d/dl)	0.45	0.21–0.95	**0.03**	0.36	0.17–0.78	**<0.01**
IFTA >25%	2.98	1.13–7.87	**0.03**	3.59	1.34–9.55	**0.01**
FSGS	0.61	0.18–2.12	0.44			
eGFR <30 ml/min per 1.73 m^2^	1.54	0.45–5.31	0.48			
Previous IS	3.48	1.15–10.49	**0.03**	4.38	1.44–13.35	**<0.01**
PLA2R-Ab ≥ 116 RU/ml	6.04	0.67–54	0.10			
Worsening kidney function and/or ESKD (*N* = 11)						
Male	1.52	0.32–7.06	0.59			
White	1.23	0.15–9.75	0.83			
Proteinuria (per 1 g/24 h)	1.0	0.92–1.1	0.83			
Serum albumin (per1 d/dl)	0.6	0.22–1.62	0.31			
IFTA >25%	2.42	0.50–11.58	0.27			
FSGS	0.84	0.18–3.92	0.83			
eGFR < 30 ml/min per 1.73 m^2^	5.23	1.32–20.68	**0.02**	3.96	0.99–15.71	0.05
Previous IS	4.55	0.98–21.08	0.05	3.87	0.82–18.29	0.08
PLA2R-Ab ≥ 116 RU/ml	1.49	0.20–10.7	0.69			
Worsening kidney function and /or Failure (*N* = 26)						
Male	1.81	0.62–5.26	0.27			
White	0.70	0.24–2.06	0.52			
Proteinuria (per 1 g/24 h)	1.03	0.98–1.08	0.15			
Serum albumin (per1 d/dl)	0.53	0.28–1.01	0.05			
IFTA > 25%	2.94	1.21–7.09	**0.02**	2.88	1.15–7.17	**0.02**
FSGS	0.80	0.30–2.12	0.65			
eGFR (per 10 ml/min per 1.73 m^2^)	0.79	0.65–0.95	**0.01**	0.85	0.71–1.02	0.09
eGFR < 30 ml/min per 1.73 m^2^	2.36	0.88–6.35	0.08			
Previous IS	3.19	1.28–7.95	**0.01**	2.95	1.16–7.52	**0.02**
PLA2R-Ab > 150 RU/ml	2.14	0.52–8.71	0.28			

CI, confidence interval; eGFR, estimated glomerular filtration rate; ESKD, end-stage kidney disease; FSFG, focal segmental glomerulosclerosis; HR, hazard ratio; IFTA, Interstitial fibrosis / tubular atrophy; IS, immunosuppression; PLA2R-Ab, Anti-PLA2R auto-antibodies; RTX, rituximab.

The bold indicates *P*-values less than 0.05, highlighting statistically significant results.

**Figure 3:**
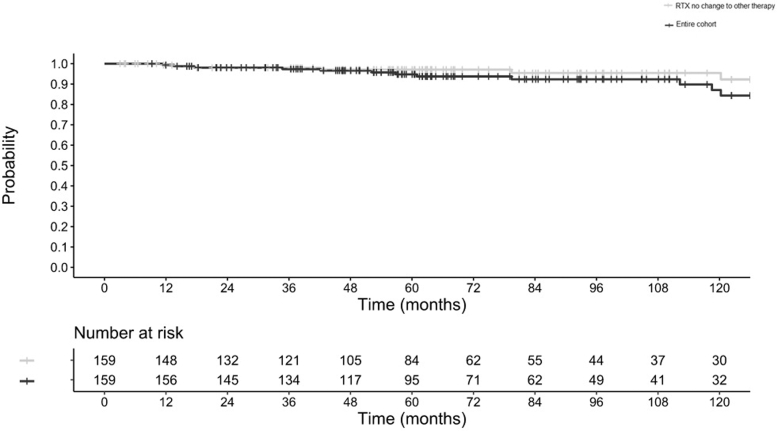
Comparative of the Kaplan Meier plot for renal survival in months in patients with MN treated only with RTX after being included versus all patients in the cohort. MN, membranous nephropathy; RTX = rituximab.

In the entire cohort, regardless of change of RTX therapy, 14 (8.8%) reached ESKD after a median follow-up of 67.6 months, and an estimated renal survival was 99% (95% CI: 98%–100%) at 1 year, 98% (95%CI: 96%–100%) at 2 years, 94.8% (95%CI: 91%–98%) at 5 years, and 87% (95% CI:78%–96%) at 10 years (Figure 3). In the multivariate Cox regression analysis, failure to respond to RTX and eGFR < 30 ml/min per 1.73 m^2^ were the main predictors of ESKD in the entire cohort (Supplementary Table S6).

In renal survival analysis for patients treated only with RTX, 7 patients (4.4%) had ESKD after a median follow-up of 62.6 (37.1–100.4) months (Supplementary Table S7). Estimated renal survival was 99% (95% CI: 98%–100%) at 1 year, 98% (95% CI: 96%–100%) at 2 and 3 years, 97% (95% CI: 94%–100%) at 5 years and 95.4% (95% CI: 91.2%–99%) at 10 years (Supplementary Figure S4). Eleven patients (6.9%) had worsening kidney function. Estimated survival free of worsening kidney function and/or ESKD was 99% (95% CI: 98%–100%) at 1 year, 97% (95% CI: 94%–99%) at 2 and 3 years, 96.4% (95% CI: 93%–99%) at 5 years, and 91% (95% CI: 84.4%–98%) at 10 years (Supplementary Figure S5).

Patients with baseline eGFR ≥ 30 ml/min per 1.73 m^2^ and no previous IS were less likely to develop ESKD and worsening kidney function (Supplementary Figures S6A and B, and S7A and B) No significant differences in renal survival were observed concerning PLA2R status, baseline PLA2R-Ab levels (≥116 U/ml vs. < 116 U/ml), nephrotic syndrome at baseline, the presence of FSGS lesion, or IFTA > 25% (log-rank *P* > 0.05).

Univariate Cox analysis identified eGFR < 30 ml/min per 1.73 m^2^ as the main predictor for worsening kidney failure (Table 2). In the multivariate Cox regression analysis of a composite outcome of failure and/or worsening kidney function, only 2 were significantly associated, namely previous IS (HR: 2.95 [95% CI: 1.16–7.52], *P* = 0.02) and IFTA ≥ 25% (HR: 2.88 [95% CI: 1.15–7.17, *P* = 0.02]) (Table 2).

In patients who responded to RTX (140/159), 4 (2.85%) developed ESKD with an estimated renal survival of 99 % (95% CI: 97%–100%) at 5 years, and 97.4 % (95% CI:94%–100%) at 10 years.

### Immunological Remission

Among the 159 patients, 16 (10.1%) had PLA2R serum status unknown, 35 (22%) were PLA2R-Ab negative, and 108 (67.9%) were PLA2R-Ab positive. Among the 108 patients with positive PLA2R-Ab, 21 patients lacked continuous monitoring of the antibody testing, and 6 never reached IR or had a clinical response (patients #3, #11, #12, #16, and #18) (Supplementary Table S5).

Time to IR was assessed through serial samples of PLA2R-Ab in 81 patients; the median time to IR was 4.9 (95% CI: 3.9–6.3) months. Among these 81 patients, 44 achieved CR, 36 achieved PR, and 1 had RP. The median (IQR) time to IR in patients with CR was 5.92 (3.49–8.88) months, 4.55 (2.36, 7.51) months in those with PR, and 8.33 months in those with RP (Kruskal-Wallis’s test *P* = 0.31) (Supplementary Table S8).

### Treatment Characteristics

The median (IQR) time between biopsy-proven MN diagnosis and initiation of RTX was 11 (4.0–29.0) months. Three treatment regimens were used for induction therapy: 1 g (day 1/day 14) in 127 (79.9%), 375 mg/m^2^ every 4 weeks in 29 (18.2%), and a single 1 g dose in 3 patients (1.9%).

One hundred sixteen patients (73%) underwent retreatment with RTX. Most patients were retreated with RTX at 6 months regardless of the CD20+ or PLA2R status, and most received 1g dose. The median (IQR) time to redose was 7.3 (6.0–14.9) months, with a median (IQR) number of treatments of 2 (1.0–3.0) per patient and a median (IQR) cumulative dose of RTX at the end of follow-up of 4 (3–6.7) g.

One hundred eleven patients (70.7%) were receiving anticoagulant or acetylsalicylic acid therapy as part of their standard treatment regimen (Table 3). Fourteen patients (8.8%) had a history of a thromboembolic event, and 4 (2.5%) presented a thromboembolic event (deep vein thrombosis), no patients developed pulmonary embolism during follow-up.

**Table 3:** Medication characteristics and adverse event profile

Induction therapy	
RTX dosing	*N* = 159
RTX regimen 1 g (day 0/day 14)	127 (79.9%)
RTX 375 mg/m^2^ weekly for 4 doses	29 (18.2%)
RTX 1 g single dose	3 (1.9%)
Re-treatment	116 (73.0%)
Number of treatments	2 (1–3)
Time to retreatment (mos)	7.3 (6–14.9)
Prophylaxis, *n* (%)	108 (67.9%)
Adverse event	37 (23.3%)
Infusion reactions	20 (12.6%)
Hypogammaglobulinemia	2 (1.3%)
Leukopenia or neutropenia	4 (2.5%)
Infection	12 (7.5%)
Respiratory tract infection	7 (58.3%)
Other therapies	
Acetylsalicylic acid	79 (71.2%)
Warfarin	15 (13.5%)
Direct oral anticoagulants	14 (12.6%)
Heparin	3 (2.7%)

IQR, interquartile range; RTX, rituximab.

Data are presented as *n* (%) or median (IQR).

### Relapses

A total of 61 of 159 patients (38.3%) relapsed. The cumulative incidence of relapse at 12, 24, and 60 months was 9.7%, 16.1%, and 33.7%, respectively. The median (IQR) time to first relapse was 36.5 (13.1–60.5) months, and the median (IQR) number of relapses per patient was 1 (1–2) (Supplementary Figure S8).

### Adverse Events

The most common side effects of RTX were infusion-related reactions, which were documented in 20 patients. Other adverse events included infections in 12 patients (predominantly respiratory tract infections in 7/12). No patient developed pneumocystis infection, although prophylaxis was used on only 108 patients (67.9%), most commonly trimethoprim-sulfamethoxazole, followed by atovaquone and pentamidine (Table 3).

Six patients (3.77%) died, with a median time to death of 50.4 (38.0–114.3) months. Two patients developed ESKD and died after > 1 year of renal replacement therapy; 1 patient had a major cardiovascular event. Two patients died from a malignancy (1 extensive stage small cell lung carcinoma and 1 high-grade urothelial carcinoma); 1 patient’s cause of death was unknown.

The incidence rate of cancer was 13.5 new cancer cases per 1000 person-years. The mean (SD) age was 70 (9.19) years. Eight (66.7%) were male, and 6 (50%) were with PLA2R-MN. Eight (66.6%) were treated with IS before, 3 (25%) with cyclophosphamide, 4 (33.3%) with cyclosporine, and 1(8.3%) with mycophenolate mofetil. The median time from RTX treatment to cancer diagnosis was 7.5 (4.04–12) years. Patients aged > 65 years had a higher cancer incidence (log-rank *P* = 0.02).

## Discussion

Although the short-term clinical efficacy of RTX in MN has been demonstrated,^5^^,^^6^^,^^8^, ^9^, ^10^^,^^16^ data on long-term outcomes are scarce; to the best of our knowledge, our study is the first to assess long-term outcomes in a population at high risk for disease progression. The main finding of the present study is the excellent long-term outcome in patients with MN treated with RTX, attaining estimated renal survival of 97% at 5 years and 95% at 10 years with a low incidence of worsening kidney function. Our cohort was at risk of progressive disease, as evidenced by high baseline proteinuria, high proportion of patients with nephrotic syndrome, reduced eGFR of 54.6 (37.4–72.5) ml/min per 1.73 m^2^ and failure to respond to previous immunosuppressive therapy. Previous trials using chlorambucil or cyclic corticosteroids/cyclophosphamide (demonstrated a 10-year kidney survival of 92% and 89%, respectively.^17^, ^18^, ^19^ This information has been used to justify the use of these treatment regimens as the only ones with long-term hard end point data. However, it should be noted that these patients enrolled in cyclic corticosteroids/cyclophosphamide studies, had milder diseases, as indicated by baseline proteinuria at around 5 to 6 g/24 h and eGFR at around 90 ml/min per 1.73 m^2^.^17^ Furthermore, patients treated with previous IS (corticosteroids or another immunosuppressive drug)^17^, ^18^, ^19^, ^20^ or patients resistant to therapy were excluded, thus representing patients at a lower risk of progression compared with our cohort. Similarly, studies that compared RTX and cyclic regimen included patients with proteinuria < 8 g/24 h, a mean eGFR of approximately 80 ml/min per 1.73 m^2^, and shorter follow-up (both 24 months).^9^^,^^16^

Previous studies document that poor response to IS is associated with poor renal survival.^21^ Historically, patients with eGFR < 30 ml/min per 1.73 m^2^ have been consistently excluded from the randomized clinical trials.^6^^,^^8^^,^^9^^,^^22^ A paucity of retrospective studies has also suggested that patients with eGFR < 45 ml/min per 1.73 m^2^ are more likely to fail to respond to RTX.^23^^,^^24^ In our cohort, 19 patients had eGFR < 30 ml/min per 1.73 m^2^, and 22 patients had IFTA ≥ 25%. Interestingly, these patients also had favorable clinical responses. This suggests that low baseline eGFR or degree of IFTA by itself should not prohibit treatment initiation. Even though patients with IFTA ≥ 25% were at higher risk of treatment failure to respond to RTX, or worsening kidney function, the probability of progression to ESKD was not affected.

The presence of FSGS lesions has been described as an independent unfavorable prognostic factor.^25^^,^^26^ In our cohort, patients with an FSGS lesion had higher proteinuria at baseline and required more time to reach CR; however, clinical response and renal survival were ultimately unaffected. This agrees with a previous study that found that both FSGS and the grade of tubulointerstitial damage did not impact clinical response.^27^ This suggests that the presence of a secondary FSGS lesion superimposed on the MN may explain a more prolonged interval between immunological and clinical response but does not affect the overall response.

Furthermore, this aligns with additional observations in our cohort. There are patients who achieve IR but continue to experience PR without ever attaining CR. In patients with PLA2R-MN, the evaluation of PLA2R-Ab is essential to distinguish residual proteinuria secondary to ongoing immunological activity versus proteinuria as a consequence of chronic and irreversible glomerular damage.^28^ The presence of residual proteinuria in the context of IR should be treated by optimizing antiproteinuric care while the patient remains in IR.^5^

Our clinical remission rate (CR or PR) was 85.7% at 2 years and 96.5% at 5 years. In the MN Trial of RTX study, patients with < 25% reduction in baseline proteinuria at 6 months were considered failure and exited the study^6^; this likely reduced the overall efficacy of therapy because clinical remissions generally follow the immune response and take longer. Indeed, our results align with the percentage of remission reported in a previous observational study, where patients were not censored at 6 months based on proteinuria response at that time, with 80% remission at 24 months,^29^ and with the RTX or Cyclophosphamide in the Treatment of MN study, which had 85% CR or PR at 24 months,^9^ even though proteinuria at baseline and PLA2R-Ab levels were higher in our cohort.

In our study, the overall incidence of treatment failure was 11.9%. In these patients, the multivariate analysis showed that previous failure to other immunosuppressive therapy or IFTA > 25% is associated with failure to respond to RTX. These patients are more likely to benefit from new anti-CD20 therapies, such as obinutuzumab, which has a more potent and prolonged B-cell depletion.^30^^,^^31^

MN is a relapsing-remitting disease, and a key target of therapy is to decrease the frequency, severity, and duration of relapses. Therefore, in patients with PLA2R-MN, diligent monitoring of anti-PLA2R-Ab is imperative to manage this condition.^13^^,^^32^ Our relapse rate was 16.1% and 33.7% at 2 and 5 years, respectively, supporting the fact that relapse rates are dependent on the duration of follow-up. A recent study of RTX 1 g (days 1 and 15) combined with low-dose cyclophosphamide (8 weeks) and prednisone (28 weeks), with RTX retreatment every 4 months for 2 years, showed a clinical response of 100% during the 38 months of median follow up. Patients who were kept on B-cell depletion did not relapse. Despite this intense IS, 10% of patients still relapsed 2 years after B cell reconstitution.^33^

Most adverse events were mild, with 37 patients (23.3%) having an event. Infusion reactions are common, as described in previous studies,^9^^,^^11^ and occur in most patients; the retrospective nature of our study is a limitation in evaluating the totality of infusion-related events. Historically, studies with cyclophosphamide are not reliable in evaluating toxicity because they were conducted before the implementation of the Guidelines for Good Clinical Practice^34^; if we look at more recent studies, the documented side effects of cyclophosphamide are significantly higher, reported as 98% in the STARMEN trial^16^ and 66% at 51 months in du Buf-Vereijken *et al.*^35^ These findings suggest that the safety profile of RTX is superior.

In our cohort, we documented that the incidence rate for cancer was 3 times higher than in the general population.^36^ Previous studies had documented that individuals with MN had higher cancer incidence, with a rate of 28 per 1000 person-years^37^ and a prevalence of 10% (95% CI: 6.1–14.6).^38^ In addition, 80% of cancer detections were after a median time of 60 months of MN diagnosis.^37^ These studies confirm that the risk of cancer is not limited to the diagnosis of MN because the risk persists for many years after the diagnosis, with a significant limitation: information about treatment or IS regimen was not provided.^38^^,^^39^ In terms of evaluating the use of RTX and the risk of cancer, previous studies did not find an excess risk in the MN population,^40^ and in other autoimmune diseases such as rheumatoid arthritis, the use of RTX was associated with an incidence rate of 7.4 cancer cases per 1000 person-years.^41^ However, our cohort has several differences. First, the majority of our population had been exposed to previous IS (7, 63.6%). Second, there is a possible incomplete report of events in previous studies because of the lack of a standardized cancer registry before 1998.^42^ Third, the longer follow-up period, the higher the number of cases. Lastly, we included the non-melanoma skin cancer in our statistics.

In contrast, our population had a mean (SD) age of 70 (9.19) years at cancer diagnosis. The cancer incidence rate in the general population aged > 65 years is 15.9 cases per 1000 person-years.^43^ Thus, our incidence is not higher when adjusted for age-specific population. Compared with other therapies, patients with MN treated with cyclophosphamide have reported a cancer incidence rate of 21.2 per 1.000 person-years,^44^ showing a higher incidence of cancer compared with our cohort. This agrees with other studies showing that RTX is associated with a lesser risk of cancer in patients with MN compared with cyclophosphamide.^40^

Our study has limitations. It is a retrospective study, and because of the clinical practice changes regarding treatment of MN at the Mayo Clinic that started in the late 1990s, we do not have a comparative group with cyclophosphamide-based therapy in a large number of patients. In 27 patients (17%) treated with RTX, follow-up was < 24 months; however, in 17 of 27 (63%%), the short follow-up was due to failure to respond to RTX or ESKD, 6 of 27 (22.2%) with RTX initiation in 2022, and only 4 of 27 (14.8%) censored for lost follow-up. Another limitation is that patients were not followed-up with using a protocol for monitoring of anti-PLA2R-Ab and B cell numbers. It is now clear that regular monitoring of anti-PLA2R-Ab levels and B cells is crucial and having a treatment goal of achieving complete IR may have further improved the excellent long-term outcomes observed in our cohort. In addition, anti-PLA2R-Ab at baseline (median 84 RU/ml) was available only in 73% of PLA2R-MN at treatment initiation, and the use of previous IS in 50% of the patients impacted anti-PLA2R levels at baseline. However, PLA2R status was available in 89.9% at some point in the follow-up. As discussed, PLA2R-Ab monitoring was also not protocolized. Nevertheless, our study has several strengths. Notably, it represents the largest cohort of patients with MN treated with RTX and with the longest follow-up. It provides valuable insights into RTX’s real-world practice and on safety over an extended period. Prolonged and repeated use of RTX in these patients appears safe, and its use is associated with excellent long-term kidney function. These findings highlight the significant benefit observed in kidney survival with RTX use, thus reinforcing the use of RTX as the preferred treatment option in patients with MN at high risk of progression.

## Disclosure

All the authors declared no competing interests.

## Data Availability Statement

Due to ethical and privacy considerations, analyses of the clinical will be available upon request to the center.

## Author Contributions

MJV-B and FCF designed the study. MJV-B, EL, YR, IR, DV, and MM abstracted the data. MJV-B and IR performed the statistical analysis. The manuscript was drafted and written by MJV-B, EL, YR, IR, MS, GMB, AC, DC, and FF. All the authors provided input for the final version of the manuscript.

## Supplementary Material

Supplementary File (PDF) **Figure S1.** Flowchart of inclusion and exclusion criteria. **Figure S2.** Cumulative incidence of clinical response (complete or partial remission), as determined by the Kaplan-Meier estimator in patients with MN receiving RTX as first line therapy by Anti-PLA2R antibodies titers. **Figure S3.** Cumulative incidence of failure, as determined by the Kaplan-Meier estimator in patients with MN receiving RTX in months by (a) all cohort. (b) Interstitial fibrosis / tubular atrophy ≥ 25% versus < 25%. (c) previous immunosuppression. **Figure S4.** Kaplan Meier plot for renal survival (95% CI in grey) in membranous nephropathy patients treated with RTX. **Figure S5.** Estimated survival (95% CI in grey) free of worsening kidney function (50% reduction in baseline eGFR) after treatment with RTX. **Figure S6.** Renal survival during follow-up in patients with MN receiving RTX in months, by a) eGFR ≥30 ml/min per 1.73 m^2^ versus eGFR < 30 ml/min per 1.73 m^2^. (b) Previous Immunosuppression yes versus no. **Figure S7.** Survival free of worsening kidney function during follow-up in patients with MN receiving RTX in months, by (A) eGFR ≥ 30 ml/min per 1.73 m^2^ versus eGFR < 30 ml/min per 1.73 m^2^. (B) Previous Immunosuppression yes versus no. **Figure S8.** Cumulative incidence of the first relapse, as determined by the Kaplan-Meier estimator in patients with MN receiving RTX in months. **Table S1.** Comparison of patient characteristics based on rituximab use: first-line therapy, relapse, and non-responder groups. **Table S2.** Clinical characteristics and clinical outcomes of patients with and without FSGS lesions. **Table S3.** Change in laboratory measures from baseline in patients with 5-year follow-up. **Table S4.** Sensitivity Analysis of Clinical Response Rates at 12, 24, and 60 months for nephrotic vs. non-nephrotic patients. **Table S5.** Clinical characteristics and clinical outcomes of patients categorized as failure. **Table S6.** Univariate and multivariate Cox regression of predictive factors for ESKD in the entire cohort of patients with membranous nephropathy treated with rituximab. **Table S7.** Clinical characteristics of patients that reached ESKD treated exclusively with Rituximab after enrollment. **Table S8.** Time to clinical and immunological response in patients with PLA2R-associated MN and availability of serial PLA2R-Ab samples. STROBE Checklist.

## Footnotes

Supplementary File (PDF)
**Figure S1.** Flowchart of inclusion and exclusion criteria. **Figure S2.** Cumulative incidence of clinical response (complete or partial remission), as determined by the Kaplan-Meier estimator in patients with MN receiving RTX as first line therapy by Anti-PLA2R antibodies titers. **Figure S3.** Cumulative incidence of failure, as determined by the Kaplan-Meier estimator in patients with MN receiving RTX in months by (a) all cohort. (b) Interstitial fibrosis / tubular atrophy ≥ 25% versus < 25%. (c) previous immunosuppression. **Figure S4.** Kaplan Meier plot for renal survival (95% CI in grey) in membranous nephropathy patients treated with RTX. **Figure S5.** Estimated survival (95% CI in grey) free of worsening kidney function (50% reduction in baseline eGFR) after treatment with RTX. **Figure S6.** Renal survival during follow-up in patients with MN receiving RTX in months, by a) eGFR ≥30 ml/min per 1.73 m^2^ versus eGFR < 30 ml/min per 1.73 m^2^. (b) Previous Immunosuppression yes versus no. **Figure S7.** Survival free of worsening kidney function during follow-up in patients with MN receiving RTX in months, by (A) eGFR ≥ 30 ml/min per 1.73 m^2^ versus eGFR < 30 ml/min per 1.73 m^2^. (B) Previous Immunosuppression yes versus no. **Figure S8.** Cumulative incidence of the first relapse, as determined by the Kaplan-Meier estimator in patients with MN receiving RTX in months. **Table S1.** Comparison of patient characteristics based on rituximab use: first-line therapy, relapse, and non-responder groups **Table S2.** Clinical characteristics and clinical outcomes of patients with and without FSGS lesions. **Table S3.** Change in laboratory measures from baseline in patients with 5-year follow-up. **Table S4.** Sensitivity Analysis of Clinical Response Rates at 12, 24, and 60 months for nephrotic vs. non-nephrotic patients. **Table S5.** Clinical characteristics and clinical outcomes of patients categorized as failure. **Table S6.** Univariate and multivariate Cox regression of predictive factors for ESKD in the entire cohort of patients with membranous nephropathy treated with rituximab. **Table S7.** Clinical characteristics of patients that reached ESKD treated exclusively with Rituximab after enrollment. **Table S8.** Time to clinical and IR in patients with PLA2R-associated MN and availability of serial PLA2R-Ab samples. STROBE Checklist.
