# Supplementary material for: Long-term Outcome of Adult Patients With Membranous Nephropathy Treated With Rituximab
Source: Kidney Int Rep. 2025 May 21;10(8):2630–41. doi: 10.1016/j.ekir.2025.05.013 (PMC12348103; doi:10.1016/j.ekir.2025.05.013)
Supplement: Supplementary File (PDF) — Figure S1. Flowchart of inclusion and exclusion criteria. Figure S2. Cumulative incidence of clinical response (complete or partial remission), as determined by the Kaplan-Meier estimator in patients with MN receiving RTX as first line therapy by Anti-PLA2R antibodies titers. Figure S3. Cumulative incidence of failure, as determined by the Kaplan-Meier estimator in patients with MN receiving RTX in months by (a) all cohort. (b) Interstitial fibrosis / tubular atrophy ≥ 25% versus < 25%. (c) previous immunosuppression. Figure S4. Kaplan Meier plot for renal survival (95% CI in grey) in membranous nephropathy patients treated with RTX. Figure S5. Estimated survival (95% CI in grey) free of worsening kidney function (50% reduction in baseline eGFR) after treatment with RTX. Figure S6. Renal survival during follow-up in patients with MN receiving RTX in months, by a) eGFR ≥30 ml/min per 1.73 m2 versus eGFR < 30 ml/min per 1.73 m2. (b) Previous Immunosuppression yes versus no. Figure S7. Survival free of worsening kidney function during follow-up in patients with MN receiving RTX in months, by (A) eGFR ≥ 30 ml/min per 1.73 m2 versus eGFR < 30 ml/min per 1.73 m2. (B) Previous Immunosuppression yes versus no. Figure S8. Cumulative incidence of the first relapse, as determined by the Kaplan-Meier estimator in patients with MN receiving RTX in months. Table S1. Comparison of patient characteristics based on rituximab use: first-line therapy, relapse, and non-responder groups. Table S2. Clinical characteristics and clinical outcomes of patients with and without FSGS lesions. Table S3. Change in laboratory measures from baseline in patients with 5-year follow-up. Table S4. Sensitivity Analysis of Clinical Response Rates at 12, 24, and 60 months for nephrotic vs. non-nephrotic patients. Table S5. Clinical characteristics and clinical outcomes of patients categorized as failure. Table S6. Univariate and multivariate Cox regression of predictive factors for ESKD in the entire coh [file mmc1.pdf]

Supplementary Tables and Figures

Supplementary Table 1 Comparison of patient characteristics based on rituximab use: first-line therapy, relapse, and non-responder groups

Supplementary Table 2 Clinical characteristics and clinical outcomes of patients with and without FSGS lesions.

Supplementary Table 3 Change in laboratory measures from baseline in patients with 5-year follow-up**.**

Supplementary Table 4 Sensitivity Analysis of Clinical Response Rates at 12, 24, and 60 Months for Nephrotic vs. Non-Nephrotic Patients.

Supplementary Table 5 Clinical characteristics and clinical outcomes of patients categorized as Failure.

Supplementary Table 6 Univariate and multivariate Cox regression of predictive factors for ESKD in the entire cohort of patients with membranous nephropathy treated with Rituximab.

Supplementary Table 7 Clinical characteristics of patients that reached ESKD treated exclusively with Rituximab after enrollment.

Supplementary Table 8 Time to clinical and immunological response in patients with PLA2R-associated MN and availability of serial PLA2R-Ab samples.

Supplementary Figure 1 Flowchart of inclusion and exclusion criteria.

Supplementary Figure 2 Cumulative incidence of clinical response (complete or partial remission), as determined by the Kaplan-Meier estimator in MN patients receiving RTX as first line therapy by Anti-PLA2R antibodies titers.

Supplementary Figure 3. Cumulative incidence of failure, as determined by the Kaplan-Meier estimator in MN patients receiving RTX in months by a) all cohort. b) Interstitial fibrosis / tubular atrophy ≥ 25% versus <25%, c) previous immunosuppression

Supplementary Figure 4 Kaplan Meier plot for renal survival (95% CI in grey) in membranous nephropathy patients treated with RTX.

Supplementary Figure 5 Estimated survival (95% CI in grey) free of worsening kidney function (50% reduction in baseline eGFR) after treatment with RTX.

Supplementary Figure 6 Renal survival during follow-up in MN patients receiving RTX in months, by a) eGFR ≥30ml/min/1.73m^2 versus eGFR <30ml/min/1.73m^2, b) Previous Immunosuppression yes versus no.

Supplementary Figure 7 Survival free of worsening kidney function during follow-up in MN patients receiving RTX in months, by a) eGFR ≥30ml/min/1.73m^2 versus eGFR <30ml/min/1.73m^2, b) Previous Immunosuppression yes versus no.

Supplementary Figure 8 Cumulative incidence of the first relapse, as determined by the Kaplan-Meier estimator in MN patients receiving RTX in months

Supplementary Table 1 Comparison of patient characteristics based on rituximab Use: first-line therapy, relapse, and non-responder groups

	**First-Line Therapy N=75**	**Relapse N=39**	**No-responders N=45**	**p-value**
**Demographics**				
**Age (years), median [IQR]**	56 [47.5, 66.5]	60 [50.5, 65.5]	54 [39, 63]	0.15
**Male sex, n (%)**	56 (74.7%)	30 (76.9%)	34 (75.6%)	*0.96*
**White race, n (%)**	64 (85.3%)	39 (100.0%)	36 (80.0%)	***0.02***
**Hypertension, n (%)**	36 (48.0%)	25 (64.1%)	31 (68.9%)	*0.06*
**PLA2R associated MN, n (%)**	N=69<br>55 (79.7%)	N=36<br>24 (66.7%)	N=38<br>29 (76.3%)	*0.33*
**Serum creatinine (mg/dl), median [IQR]**	1.4 [1.0, 1.8]	1.7 [1.4, 2.2]	1.5 [1.1, 1.8]	***0.004***
**eGFR (ml/min/1.73m^2), median [IQR]**	61[39.8, 75.0]	41.4 [31.2, 55.9]	54.8 [39.5, 78.4]	***0.003***
**Proteinuria (g/24hr), median [IQR]**	9.0 [7.0, 11.4]	9.4 [5.9, 12.8]	9.3 [7.3, 12.0]	*0.82*
**Albumin(g/dL), median [IQR]**	2.6 [2.2, 3.1]	3.1 [2.6, 3.4]	2.7 [2.1, 3.2]	***0.004***
**Anti PLA2R antibody titters available at the time of RTX initiation (RU/mL),] in PLA2R associated MN**	N=42<br>87[40.8, 199.5]	N=18<br>29[15, 99]	N=19<br>162[67.5, 244.5]	***0.003***

IQR: interquartile range, eGFR: estimated glomerular filtration rate, PLA2R: phospholipase A2 receptor, MN: membranous nephropathy, RTX: Rituximab.

Supplementary Table 2 Clinical characteristics and clinical outcomes of patients with and without FSGS lesions.

	MN No FSGS lesion N=119	MN and FSGS lesion N=36	p-value
**Demographics**			
**Age (years), median [IQR]**	58[46.5,65]	59[48,67]	0.61
**Male sex, n (%)**	88 (73.9%)	29 (80.6%)	*0.41*
**White race, n (%)**	104 (87.4%)	31 (86.1%)	*0.78*
**Body Mass Index (Kg/m²), mean (SD)**	29.17 [26.30, 32.87]	30.68 [27.41, 33.42]	*0.10*
**Clinical characteristics**			
**PLA2R associated MN, n (%)**	N=109<br>81(74.3%)	N=33<br>26(78.8%)	*0.60*
**Serum creatinine (mg/dl), median [IQR]**	1.45 [1.10, 1.90]	1.56 [1.20, 2.03]	*0.18*
**eGFR (ml/min/1.73m²), median [IQR]**	54.56 [38.60, 75.05]	49.25 [34.00, 67.35]	*0.19*
**Proteinuria (g/24hr), median [IQR]**	8.75 [6.56, 11.32]	10.84 [6.71, 17.45]	*0.057*
**Albumin(g/dL), median [IQR]**	2.70 (2.25, 3.20)	2.75 (2.18, 3.23)	*0.88*
**Clinical outcomes**			
**Time to first CR (months), median [IQR]**	N=60<br>18.44 [13.66, 37.06]	N=15<br>35.57 [25.40, 40.39]	***0.03***
**Time to first PR (months), median [IQR]**	N=103<br>6.28 [3.63, 11.56]	N=21<br>9.37 [3.79, 18.38]	*0.10*
**Time to first IR (months), median [IQR]**	N=59<br>4.52 [2.81, 9.52]	N=23<br>5.98 [3.44, 8.61]	*0.79*

SD: standard deviation, IQR: interquartile range, eGFR: estimated glomerular filtration rate, PLA2R: phospholipase A2 receptor, MN: membranous nephropathy, CR: Complete Remission, PR: Partial remission, IR: immunological Remission.

Supplementary Table 3 Change in laboratory measures from baseline in patients with 5 years follow up.

	N	Median [IQR]	Median Difference [95%CI]	p-value
**Serum creatinine (mg/dl)**				
Baseline	85	1.50[1.1,1.9]	-	-
12 months – baseline	85	1.30[1.0,1.66]	-0.20 [-0.27, -0.12]	***<0.001***
24 months - baseline	85	1.26[1.0.1.65]	-0.22[-0.32, -0.12]	***<0.001***
60 months- baseline	85	1.20[1.00,1.63]	-0.22 [-0.32, -0.13]	***<0.001***
**eGFR (ml/min/1.73m²)**				
Baseline	85	54.56 [37.3,72.27]	-	**-**
12 months – baseline	85	60.82[44.67,86.66]	8.19 [5.26,11.09]	***<0.001***
24 months – baseline	85	63.70[47.20, 86.12]	8.64[4.85,12.49]	***<0.001***
60 months – baseline	85	67.43[45.03, 81.87]	7.19 [4.01,11.12]	***<0.001***
**Proteinuria (g/24h)**				
Baseline	85	8.75[6.68,11.23]	-	**-**
12 months – baseline	85	1.82 [0.74, 3.14]	-7.34 [-8.31, -6.48]	***<0.001***
24 months – baseline	85	0.84 [0.29, 1.97]	-7.80 [-8.96, -6.82]	***<0.001***
60 months – baseline	85	0.50 [0.15, 1.43]	-7.77[-9.48, -7.45]	***<0.001***

IQR: interquartile range, CI: Confidence interval, eGFR: estimated glomerular filtration rate.

Supplementary Table 4 Sensitivity Analysis of Clinical Response Rates at 12, 24, and 60 Months for Nephrotic vs. Non-Nephrotic Patients

	Overall Response	No Nephrotic Syndrome N=23(14.5%)	Nephrotic Syndrome N=136(85.5%)	P value
**12 months (n=149)**				0.22
**CR**	14 (9.4%)	3 (13.0%)	11 (8.7%)	
**PR**	99 (66.4%)	18 (78.3%)	81 (64.3%)	
**24 months (n=133)**				0.54
**CR**	42 (31.6%)	8 (38.1%)	34 (30.4%)	
**PR**	72 (54.1%)	12 (57.1%)	60 (53.6%)	
**60 months (n=85)**				0.87
**CR**	35 (41.2%)	5 (38.5%)	30 (41.7%)	
**PR**	47 (55.3%)	8 (61.5%)	39 (54.2%)	

Supplementary Table 5 Clinical characteristics and clinical outcomes of patients categorized as therapy Failure.

#Case	PIS	Creatinine (mg/dL)	eGFR (ml/min/1.73m^2)	Proteinuria (g/24h)	Albumin (g/dL)	PLA2R-associated MN	PLA2Rab titters (RU/mL)	Time of Follow-up in the RTX cohort (months)	RTX regimen	New Therapy	Outcome (Follow-up available after failure to RTX)
**Failure**											
**1**	Cp	1.4	62.3	8.2	3.6	Yes	NA	12	375 mg/m^2 weekly for four doses X2	ACTH	ESKD after censoring (after 118 months)
**2**	CE	1.3	62.4	10.6	2.8	NA	NA	16.14	1g (day 0/day 14) X2	CsA	ESKD after censoring (56.88 months)
**3**	Cp	2.05	33.7	12.5	2.3	Yes	25	8.5	1g (day 0/day 14) X 1	OBZ	No ESKD (Follow up 46.8 months)
**4**	No	1.45	38.3	7.08	2.9	No	Neg	11.51	1g (day 0/day 14) X2	Cp	ESKD after censoring (34.68 months)
**5**	CsA	1.9	46.2	32.6	2	No	Neg	21.00	1g (day 0/day 14) X2	Cp	No ESKD (Follow up 82.3 months)
**6**	Cp	2.5	29.2	20.08	2	NA	NA	11.97	1g (day 0/day 14) X2	No	No ESKD (Follow up 11.97)
**7**	No	2	33.5	9.62	2.1	NA	NA	5.98	375 mg/m^2 weekly	Cp	No ESKD (Follow-up 131.7 months)

									for four doses X1		Death
**8**	CE	1.8	48.80	23.44	2.1	Yes	NA	18.02	1g (day 0/day 14) X2	Cp	ESKD after censoring (214 months)
**9**	MMF	1.8	45.3	16.6	1.4	NA	NA	17.52	1g (day 0/day 14) X2	No	ESKD not censored (after 17.52 months)
**10**	Cp	1.8	47.87	13.7	2.7	No	Neg	55.6	375 mg/m^2 weekly for four doses X2 1g (day 0/day 14) X2	ACTH	ESKD after censoring (112.32months)
**11**	CsA Cp	3.93	18.18	10.80	2.6	Yes	117	11.34	1g (day 0/day 14) X1	No	ESKD not censored (11.34 months)
**12**	CsA Cp	1	86.12	15.72	2.4	Yes	125	13.15	1g (day 0/day 14) X1	OBZ	No ESKD (Follow-up 76.7 months)
**13**	CsA	1.34	58.39	5.30	3	Yes	162	6.51	1g (day 0/day 14) X1	Cp	No ESKD (Follow-up 67 months)
**14**	CsA	1.58	59.20	11.63	2.3	No	Neg	4.11	1g (day 0/day 14) X1	Cp	ESKD after censoring (52 months)
**15**	CsA	1.9	41.64	17.83	2.3	Yes	NA	13.58	1g (day 0/day 14) X1	No	ESKD not censored

**16**	No	2.1	37.16	9.22	2.1	Yes	1500	3.88	1g (day 0/day 14) X1	Cp	No ESKD (Follow-up 94.09 months)
**17**	TAC MMF	1.3	77.66	19.79	1.6	Yes	NA	31.86	1g (day 0/day 14) X2	Cp	ESKD after censoring (61 months)
**18**	No	2.2	26.48	8.64	3.3	Yes	312	10.16	1g (day 0/day 14) X1<br><br>1g (single dose) X1	OBZ	No ESKD (Follow-up 92.55 months)
**19**	TAC	1.55	54.84	8.00	2.7	Yes	NA	3.02	1g (day 0/day 14) X1	OBZ	No ESKD (Follow-up 104.78 months)

PIS: previous Immunosuppression, eGFR: estimated glomerular filtration rate, PLA2R: phospholipase A2 receptor, MN: membranous nephropathy, FU: follow-up, RTX: rituximab, CE: corticosteroids, CsA: cyclosporine, TAC: tacrolimus, Cp: cyclophosphamide, ACTH: adrenocorticotropic hormone, OBZ: Obinutuzumab, ESKD: end-stage kidney disease. *Not censored or change therapy, NA: not applicable

Supplementary Table 6 Univariate and multivariate Cox regression of predictive factors for ESKD in the entire cohort of patients with membranous nephropathy treated with Rituximab.

ESKD (n=14)						
**Univariate**				**Multivariate**		
	**HR**	**95% CI**	**p-value**	**HR**	**(95% CI)**	**p-value**
**Serum creatinine**	3.04	1.60-5.78	**<0.001**			
**eGFR (per10ml/min/1.73m^2)**	0.96	0.93-0.99	**0.02**			
**Failure to RTX**	21.8	6.77-70.33	**<0.001**	22.6	6.97-73.01	**<0.001**
**IFTA (per 1%)**	1.04	1.01-1.09	**0.03**			
**IF/TA ≥25%**	3.84	1.26-11.65	**0.02**			
**FSGS**	0.61	0.13-2.77	0.53			
**eGFR <30 ml/min/1.73m^2**	4.30	1.13-16.2	**0.03**	4.86	1.23-19.8	**<0.001**
**Proteinuria (per 1g/24h)**	1.02	0.94-1.1	0.56			
**Previous IS**	11.79	1.53-90.3	**0.02**			
**PLA2Rab ≥ 116 RU/mL**	2.88	0.26-31.8	0.38			

eGFR: estimated glomerular filtration rate, IF/TA: Interstitial fibrosis / tubular atrophy, FSFG: focal segmental glomerulosclerosis, IS: immunosuppression, PLA2Rab: Anti-PLA2R auto-antibodies, RTX: Rituximab, ESKD: End-stage kidney disease, HR: Hazard ratio, CI: Confidence Interval.

Supplementary Table 7 Clinical characteristics of patients that reached ESKD treated only Rituximab after enrollment.

#Case	PIS	Creatinine (mg/dL)	eGFR (ml/min/1.73m^2)	Proteinuria (g/24h)	Albumin (g/dL)	PLA2R-associated MN	PLA2Rab titters (RU/mL)	RTX regimen	Clinical or immunological response	Time of Follow-up (months) to ESKD	Cause ESKD
**9**	MMF	1.8	45.3	16.6	1.4	NA	NA	1g (day 0/day 14) X2	No	17.52	No remission progression to ESKD
**11**	CsA Cp	3.93	18.18	10.80	2.6	Yes	117	1g (day 0/day 14) X1	No	11.34	No remission progression to ESKD
**15**	CsA	1.9	41.64	17.83	2.3	Yes	Neg	1g (day 0/day 14) X1	No	13.58	AKI (new biopsy interstitial nephritis)
**20**	Cp	2.8	25.19	16.5	2.2	Yes	NA	1g (day 0/day 14) X6	PR and IR	79.73	Partial nephrectomy (papillary renal cell carcinoma) AKI (pyelonephritis)
**21**	CsA Cp	1.8	32.06	10.31	2.3	Yes	NA	1g (day 0/day 14) X3<br>1g (single dose) X4	PR and IR	120.26	Progression after 10 years
**22**	Cp	2.3	33.3	4.9	3.7	No	Neg	375 mg/m2 weekly for four doses X2 1g single dose X1	PR	147.62	AKI (diverticulitis)

**23**	CsA	2.4	29.20	3.96	2.0	Yes	904	1g (day 0/day 14) X3	PR and IR	42.15	AKI (High dose NSAIDS)

PIS: previous Immunosuppression, CsA: cyclosporine, Cp: cyclophosphamide, eGFR: estimated glomerular filtration rate, PLA2R: phospholipase A2 receptor, MN: membranous nephropathy, RTX: rituximab, PR: partial response, IR: immunological remission, ESKD: end-stage kidney disease, AKI: acute kidney injury.

Supplementary Table 8 Time to clinical and immunological response in patients with PLA2R-associated MN and availability of serial PLA2R-Ab samples.

	N=81	Time to CR (months)	Time to PR (months)	Time to IR (months)
**CR**	44	23.98 [15.20, 36.86]	5.09 [3.06, 7.72]	5.92 [3.49, 8.88]
**PR**	36	No	9.64[4.15, 15.78]	4.55 [2.36, 7.51]
**RP**	1	No	No	8.33

CR: complete response, PR: partial response, RP reduction of proteinuria, IQR interquartile range. Data are presented as median [IQR].

Supplementary Figure 1 Flowchart of inclusion and exclusion criteria.

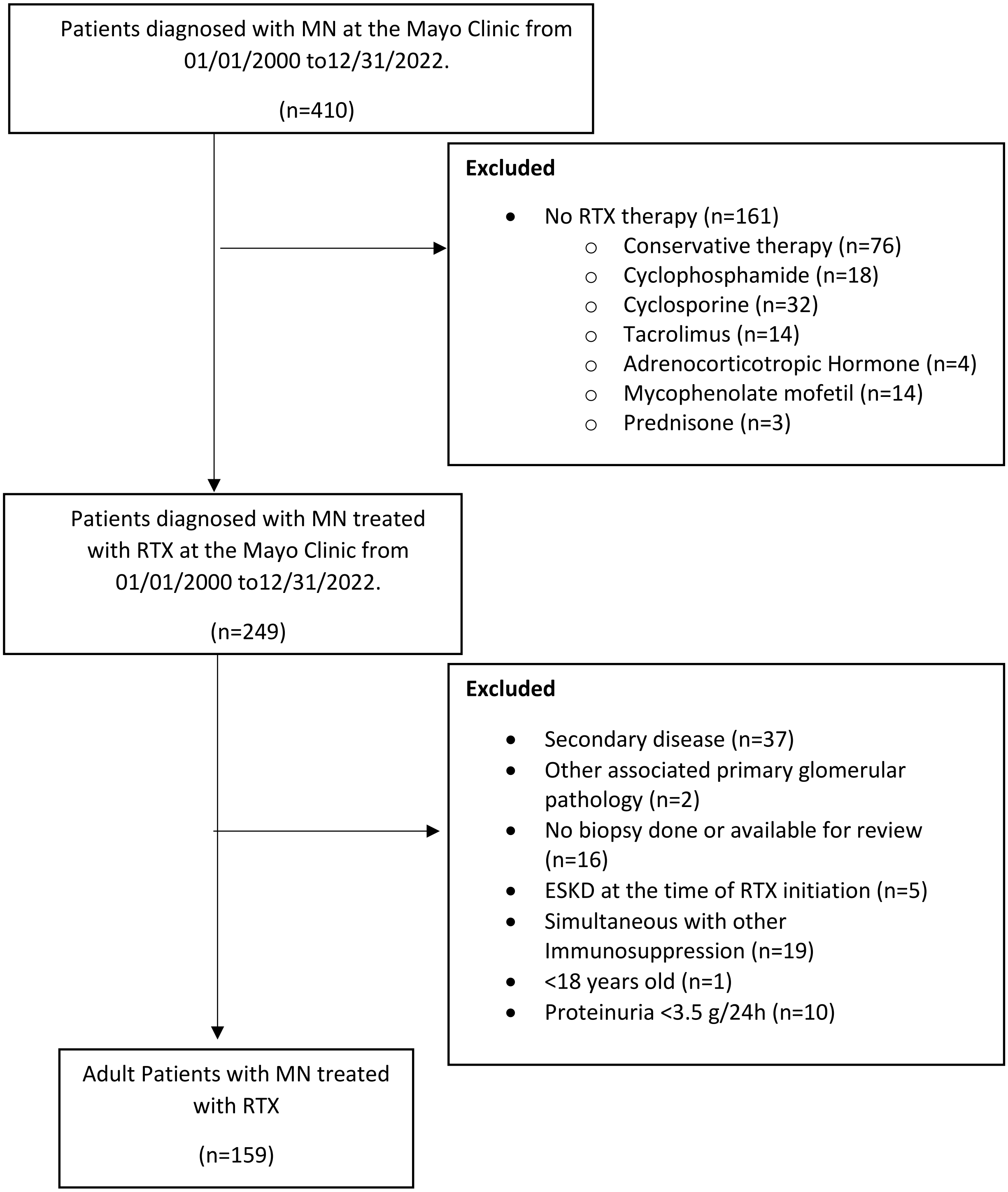

MN: Membranous Nephropathy, RTX: Rituximab, ESKD: End-stage kidney [illegible]

Supplementary Figure 2 Cumulative incidence of clinical response (complete or partial remission), as determined by the Kaplan-Meier estimator in MN patients receiving RTX as first line therapy by Anti-PLA2R antibodies titers.

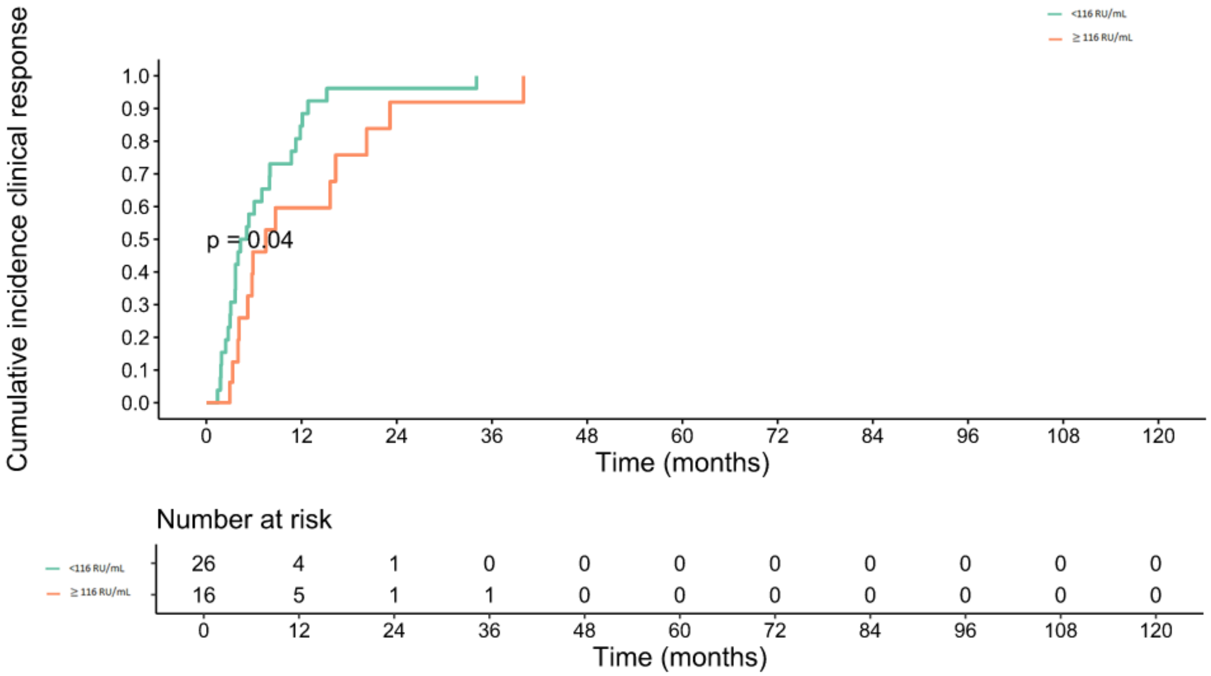

Supplementary Figure 3. Cumulative incidence of failure, as determined by the Kaplan-Meier estimator in MN patients receiving RTX in months by a) all cohort. b) Interstitial fibrosis / tubular atrophy ≥ 25% versus <25%, c) previous immunosuppression

a)

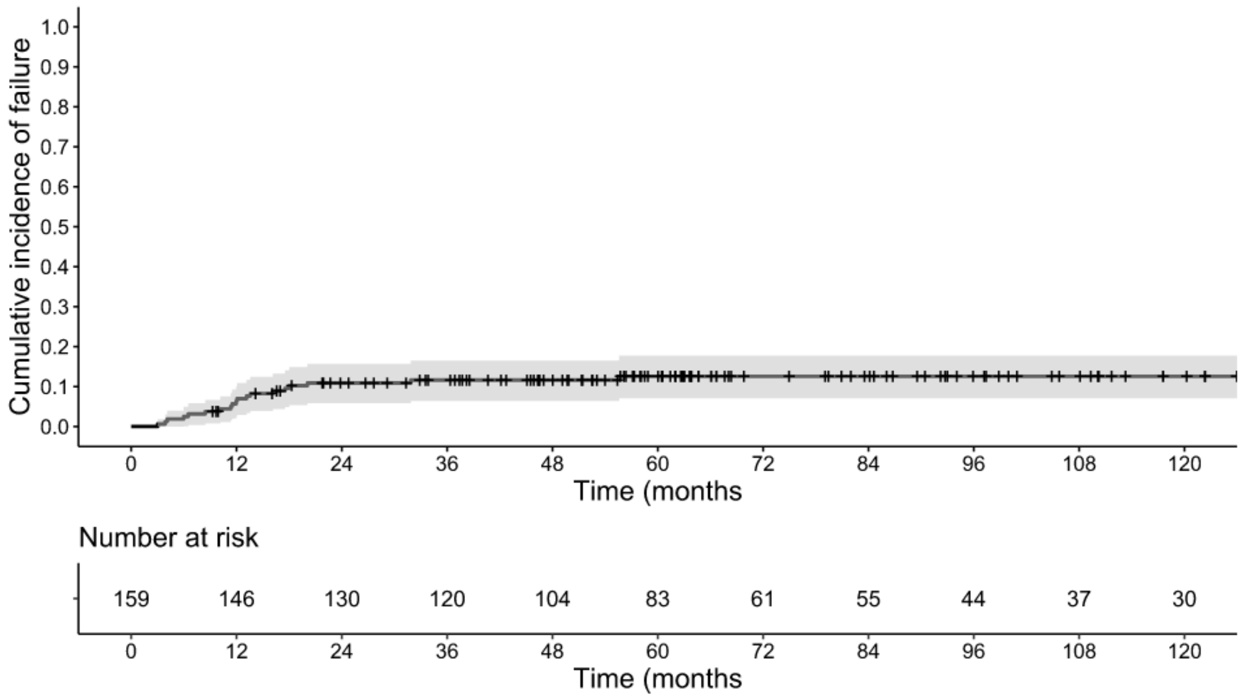

b)

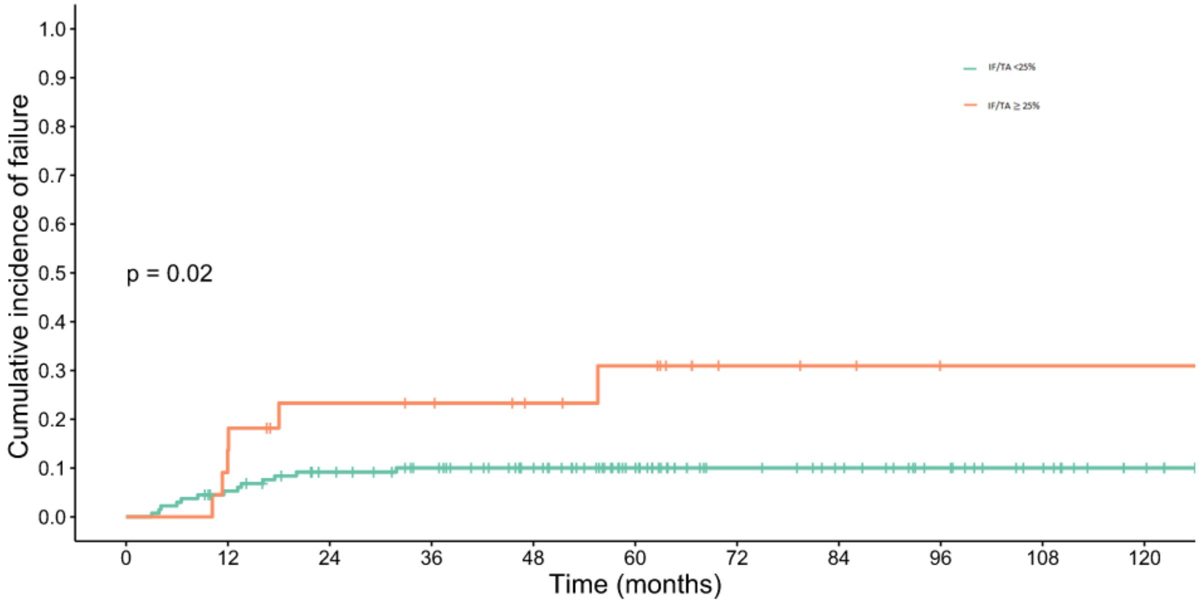
IF/TA <25%
IF/TA ≥ 25%
p = 0.02
Cumulative incidence of failure
1.0
0.9
0.8
0.7
0.6
0.5
0.4
0.3
0.2
0.1
0.0
0
12
24
36
48
60
72
84
96
108
120
Time (months)

c)

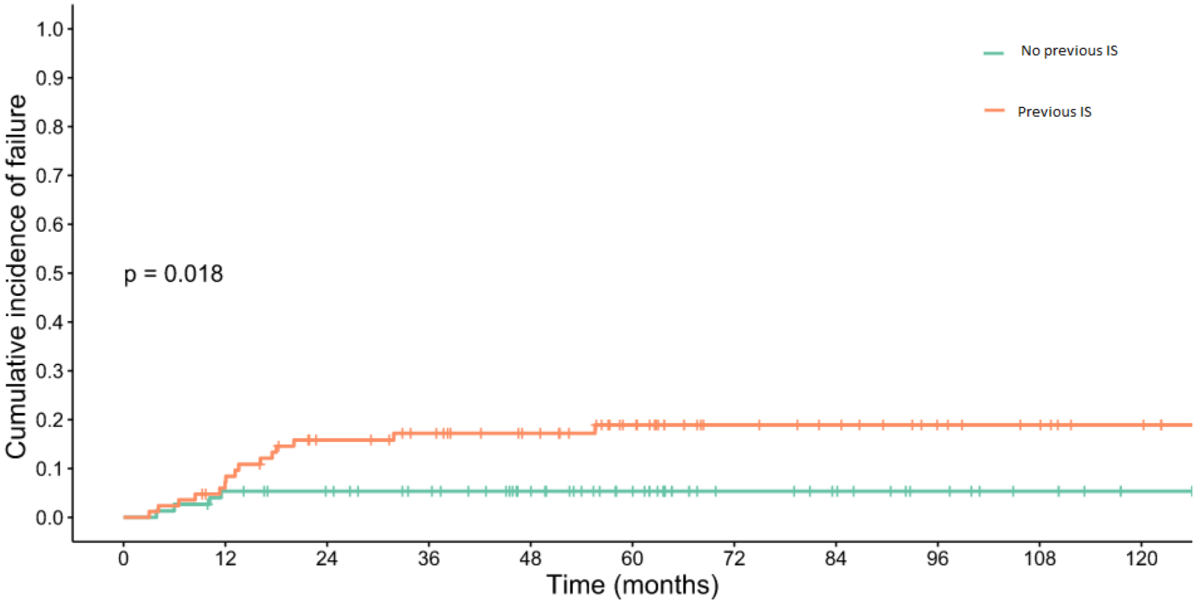
No previous IS
Previous IS
p = 0.018
Cumulative incidence of failure
1.0
0.9
0.8
0.7
0.6
0.5
0.4
0.3
0.2
0.1
0.0
0
12
24
36
48
60
72
84
96
108
120
Time (months)

Supplementary Figure 4 Kaplan Meier plot for renal survival (95% CI in grey) in membranous nephropathy patients treated with RTX.

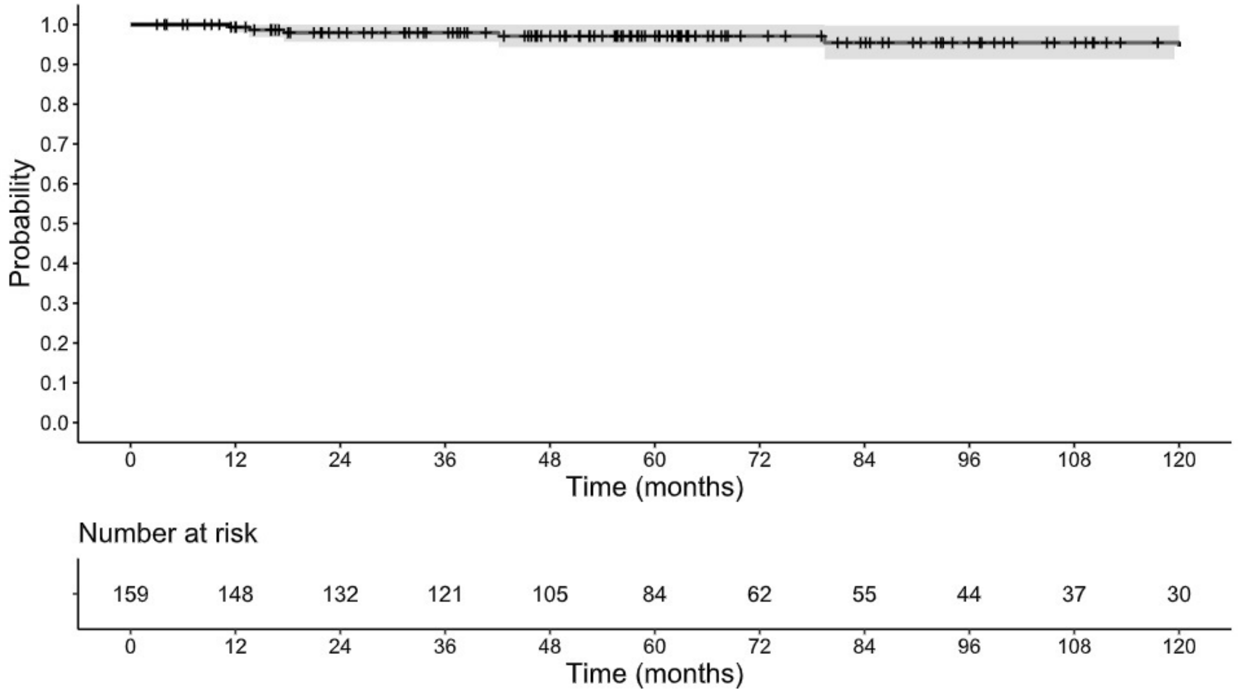

Supplementary Figure 5. Estimated survival (95% CI in grey) free of worsening kidney function (50% reduction in baseline eGFR and /or ESKD) after treatment with RTX.

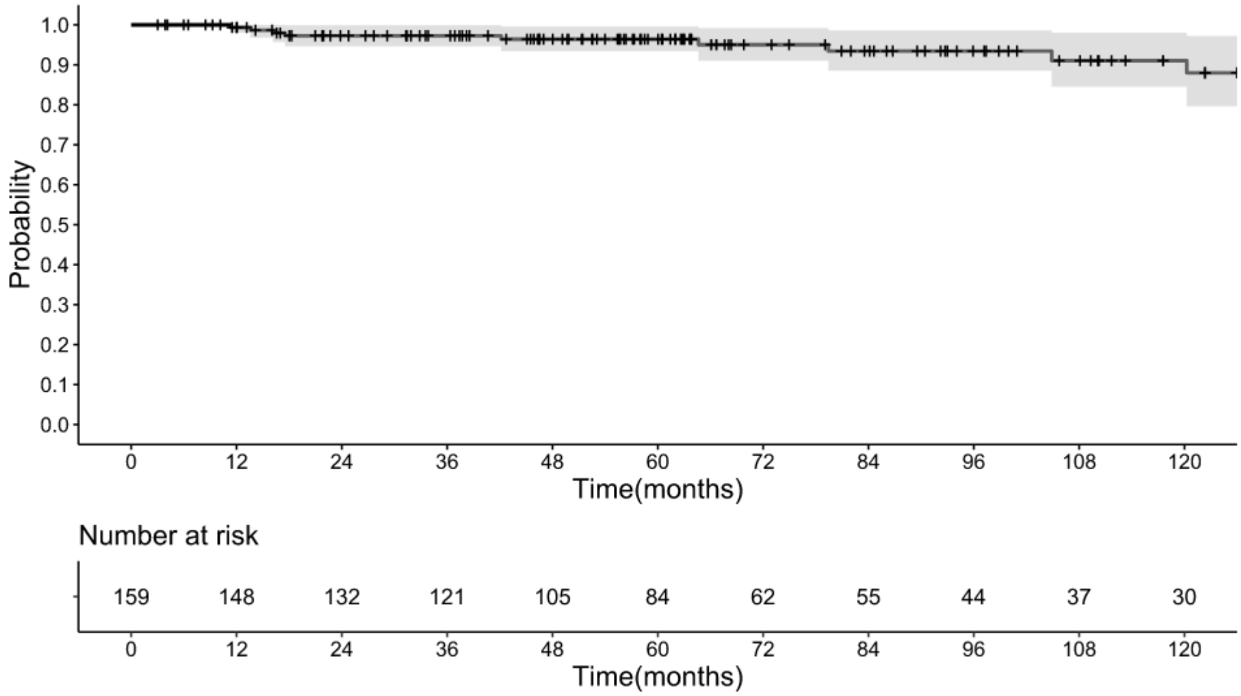

Supplementary Figure 6 Renal survival during follow-up in MN patients receiving RTX in months, by a) eGFR ≥30ml/min/1.73m^2 versus eGFR <30ml/min/1.73m^2, b) Previous Immunosuppression yes versus no.

a)

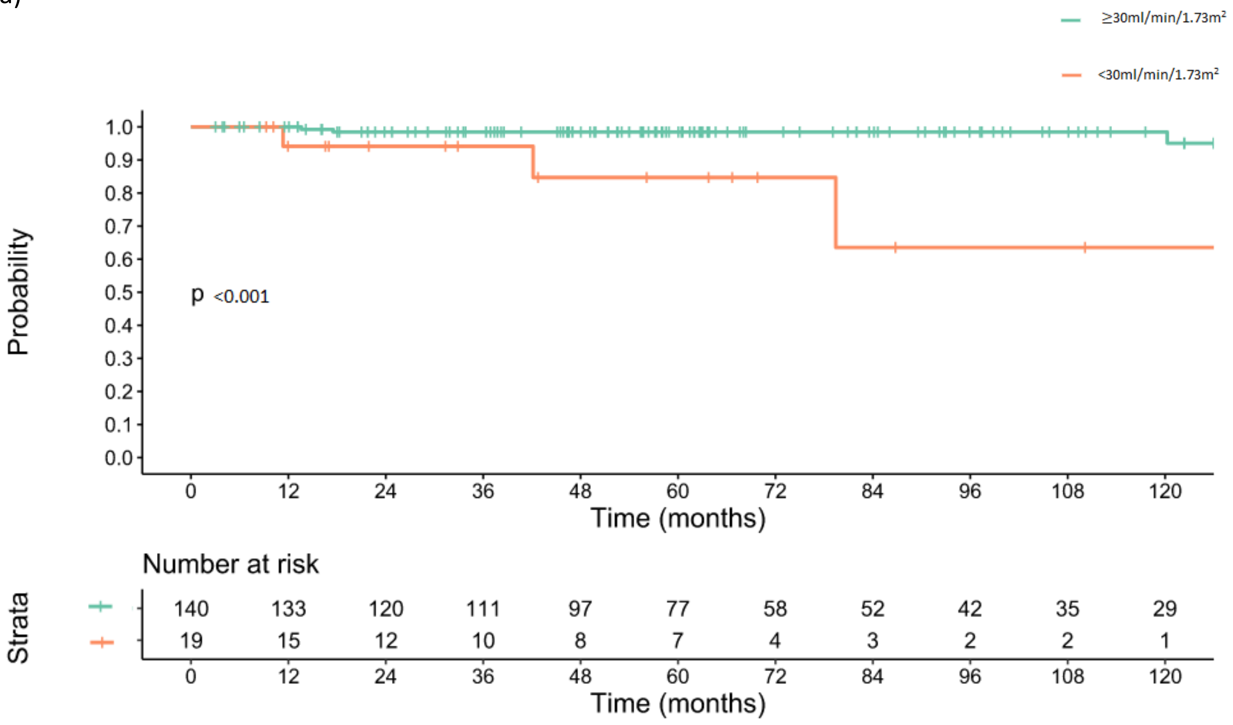

b)

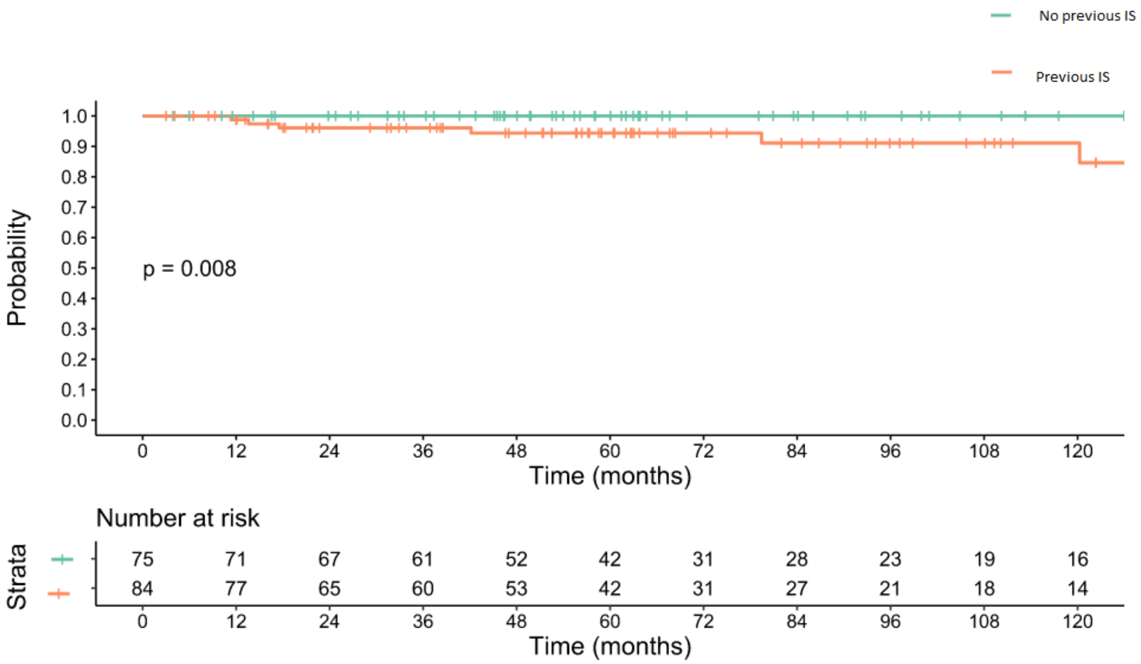

Supplementary Figure 7 Survival free of worsening kidney function during follow-up in MN patients receiving RTX in months, by a) eGFR ≥30ml/min/1.73m^2 versus eGFR <30ml/min/1.73m^2, b) Previous Immunosuppression yes versus no.

a)

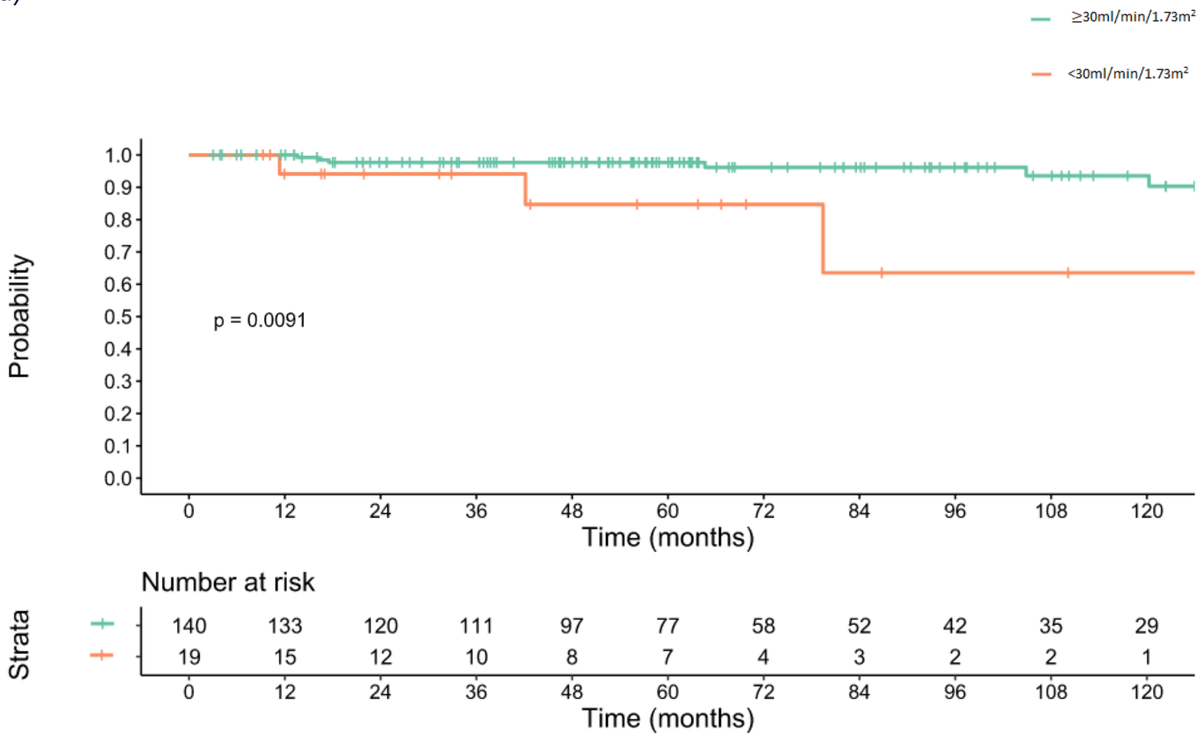

b)

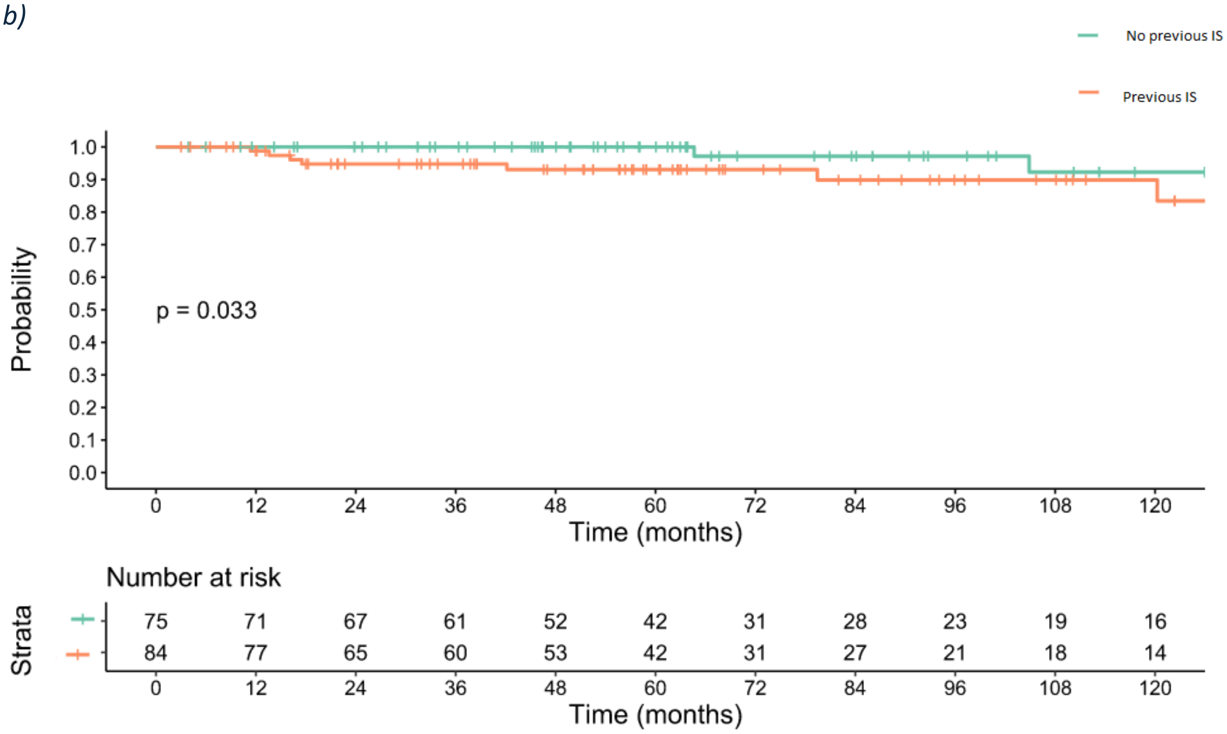

Supplementary Figure 8. Cumulative incidence of the first relapse, as determined by the Kaplan-Meier estimator in MN patients receiving RTX in months

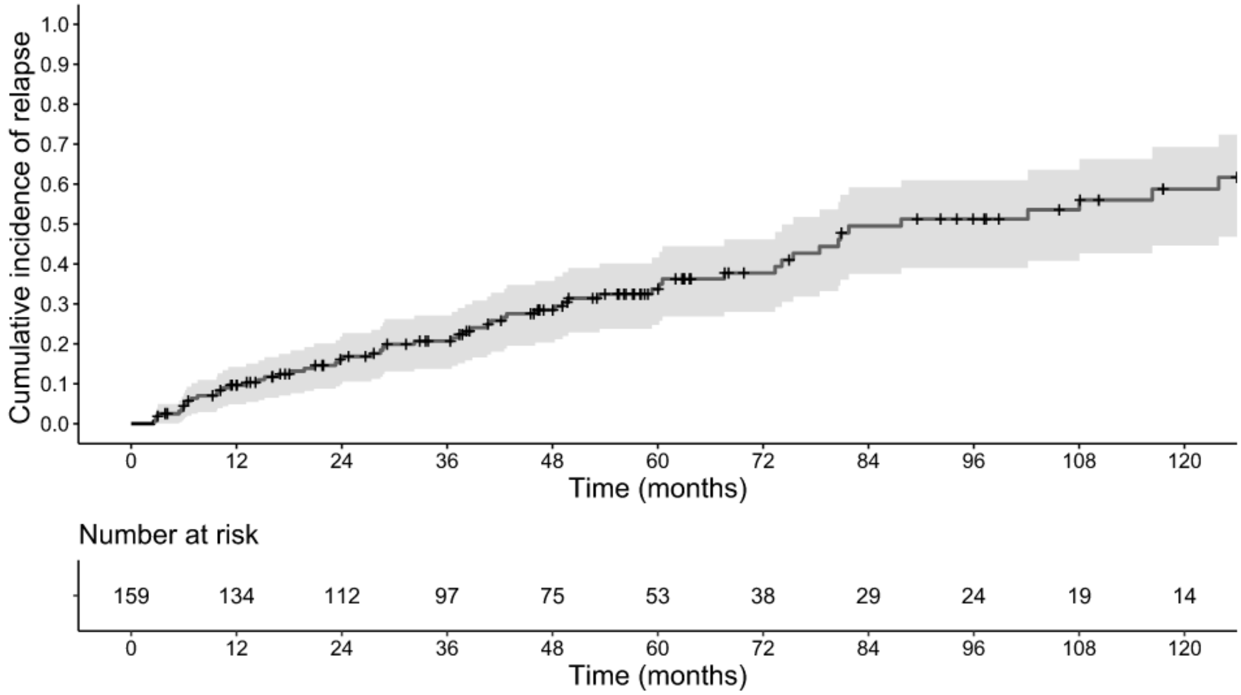

STROBE Statement—Checklist of items that should be included in reports of ***cohort studies***

	Item No	Recommendation	Page No
**Title and abstract**	1	(*a*) Indicate the study's design with a commonly used term in the title or the abstract<br>(*b*) Provide in the abstract an informative and balanced summary of what was done and what was found	2
**Introduction**			
Background/rationale	2	Explain the scientific background and rationale for the investigation being reported	3
Objectives	3	State specific objectives, including any prespecified hypotheses	3
**Methods**			
Study design	4	Present key elements of study design early in the paper	4
Setting	5	Describe the setting, locations, and relevant dates, including periods of recruitment, exposure, follow-up, and data collection	4
Participants	6	(*a*) Give the eligibility criteria, and the sources and methods of selection of participants. Describe methods of follow-up<br>(*b*) For matched studies, give matching criteria and number of exposed and unexposed	4-5<br>NA
Variables	7	Clearly define all outcomes, exposures, predictors, potential confounders, and effect modifiers. Give diagnostic criteria, if applicable	5
Data sources/ measurement	8*	For each variable of interest, give sources of data and details of methods of assessment (measurement). Describe comparability of assessment methods if there is more than one group	5-7
Bias	9	Describe any efforts to address potential sources of bias	4-7 Subgroup analysis with FSGS Sensitivity analysis for normal serum albumin patients
Study size	10	Explain how the study size was arrived at	NA
Quantitative variables	11	Explain how quantitative variables were handled in the analyses. If applicable, describe which groupings were chosen and why	6
Statistical methods	12	(*a*) Describe all statistical methods, including those used to control for confounding<br>(*b*) Describe any methods used to examine subgroups and interactions<br>(*c*) Explain how missing data were addressed<br>(*d*) If applicable, explain how loss to follow-up was addressed<br>(*e*) Describe any sensitivity analyses	6-7
**Results**			
Participants	13*	(a) Report numbers of individuals at each stage of study—eg numbers potentially eligible, examined for eligibility, confirmed eligible, included in the study, completing follow-up, and analysed<br>(b) Give reasons for non-participation at each stage<br>(c) Consider use of a flow diagram	7 Supplementary Figure 1

Descriptive data	14*	(a) Give characteristics of study participants (eg demographic, clinical, social) and information on exposures and potential confounders<br>(b) Indicate number of participants with missing data for each variable of interest<br>(c) Summarise follow-up time (eg, average and total amount)	7<br>Table 1 with missing data reporter per variable<br>*Follow-up time (page 11)
Outcome data	15*	Report numbers of outcome events or summary measures over time	Supplementary table 3 over time<br>Figure 1

Main results	16	(*a*) Give unadjusted estimates and, if applicable, confounder-adjusted estimates and their precision (eg, 95% confidence interval). Make clear which confounders were adjusted for and why they were included<br>(*b*) Report category boundaries when continuous variables were categorized<br>(*c*) If relevant, consider translating estimates of relative risk into absolute risk for a meaningful time period	7-11
Other analyses	17	Report other analyses done—eg analyses of subgroups and interactions, and sensitivity analyses	*Subgroup page 7, supplementary table 1, supplementary Table 2<br>*Sensitivity analyses, page 9 and Supplementary table 4<br>*Relapse page 12<br>*Adverse events: Safety page 12-13
**Discussion**			
Key results	18	Summarise key results with reference to study objectives	13 -16
Limitations	19	Discuss limitations of the study, taking into account sources of potential bias or imprecision. Discuss both direction and magnitude of any potential bias	17
Interpretation	20	Give a cautious overall interpretation of results considering objectives, limitations, multiplicity of analyses, results from similar studies, and other relevant evidence	17-18
Generalisability	21	Discuss the generalisability (external validity) of the study results	17-18
**Other information**			
Funding	22	Give the source of funding and the role of the funders for the present study and, if applicable, for the original study on which the present article is based	18

*Give information separately for exposed and unexposed groups.

Note: An Explanation and Elaboration article discusses each checklist item and gives methodological background and published examples of transparent reporting. The STROBE checklist is best used in conjunction with this article (freely available on the Web sites of PLoS Medicine at http://www.plosmedicine.org/, Annals of Internal Medicine at http://www.annals.org/, and Epidemiology at http://www.epidem.com/). Information on the STROBE Initiative is available at http://www.strobe-statement.org.
